# *Azotobacter* Genomes: The Genome of *Azotobacter chroococcum* NCIMB 8003 (ATCC 4412)

**DOI:** 10.1371/journal.pone.0127997

**Published:** 2015-06-10

**Authors:** Robert L. Robson, Robert Jones, R. Moyra Robson, Ariel Schwartz, Toby H. Richardson

**Affiliations:** 1 School of Biological Sciences, University of Reading, Whiteknights, Reading, United Kingdom; 2 Craic Computing LLC, Seattle, Washington, United States of America; 3 Synthetic Genomics, La Jolla, California, United States of America; Illinois Institute of Technology, UNITED STATES

## Abstract

The genome of the soil-dwelling heterotrophic N_2_-fixing Gram-negative bacterium *Azotobacter chroococcum* NCIMB 8003 (ATCC 4412) (Ac-8003) has been determined. It consists of 7 circular replicons totalling 5,192,291 bp comprising a circular chromosome of 4,591,803 bp and six plasmids pAcX50a, b, c, d, e, f of 10,435 bp, 13,852, 62,783, 69,713, 132,724, and 311,724 bp respectively. The chromosome has a G+C content of 66.27% and the six plasmids have G+C contents of 58.1, 55.3, 56.7, 59.2, 61.9, and 62.6% respectively. The methylome has also been determined and 5 methylation motifs have been identified. The genome also contains a very high number of transposase/inactivated transposase genes from at least 12 of the 17 recognised insertion sequence families. The Ac-8003 genome has been compared with that of *Azotobacter vinelandii* ATCC BAA-1303 (Av-DJ), a derivative of strain O, the only other member of the *Azotobacteraceae* determined so far which has a single chromosome of 5,365,318 bp and no plasmids. The chromosomes show significant stretches of synteny throughout but also reveal a history of many deletion/insertion events. The Ac-8003 genome encodes 4628 predicted protein-encoding genes of which 568 (12.2%) are plasmid borne. 3048 (65%) of these show > 85% identity to the 5050 protein-encoding genes identified in Av-DJ, and of these 99 are plasmid-borne. The core biosynthetic and metabolic pathways and macromolecular architectures and machineries of these organisms appear largely conserved including genes for CO-dehydrogenase, formate dehydrogenase and a soluble NiFe-hydrogenase. The genetic bases for many of the detailed phenotypic differences reported for these organisms have also been identified. Also many other potential phenotypic differences have been uncovered. Properties endowed by the plasmids are described including the presence of an entire aerobic corrin synthesis pathway in pAcX50f and the presence of genes for retro-conjugation in pAcX50c. All these findings are related to the potentially different environmental niches from which these organisms were isolated and to emerging theories about how microbes contribute to their communities.

## Introduction

Members of the genus *Azotobacter* are free-living obligately-aerobic nitrogen-fixing heterotrophic Gram-negative bacteria in the class γ-proteobacteria. *A*. *chroococcum* was the first species described [1] and the nomenclatural type species of the genus. Other species isolated subsequently include *A*. *vinelandii* [2], *A*. *beijerinckii* [3], *A*. *nigricans* [4], *A*. *armeniacus* [5] and, more recently *A*. *salinestris* [6]. A detailed taxonomic and ecological study of the *Azotobacteraceae* examining many morphological, physiological and biochemical properties of the aerobic N_2_-fixers was conducted by Thompson & Skerman [7]. The taxonomic position of Azotobacters has been reassessed using nucleotide sequence comparisons leading to the reassignment of Azotobacters to the family *Pseudomonadaceae* [8] and the genus *Pseudomonas* and within the clade including *P*. *aeruginosa* and *P*. *resinovorans* [9, 10].

Azotobacters display a number of interesting characteristics including the formation of desiccation-resistant cysts [11], the ability to develop very high respiratory rates, to produce extracellular polysaccharides such as alginates, levans, and cellulose and to accumulate poly-hydroxybutyrate. Azotobacters are generally easy to cultivate and consequently have been employed as “work horses” in studies of some fundamental biochemical processes e.g. RNA-polymerase [12], electron transport, and iron storage. Studies in *A*.*vinelandii* and *A*. *chroococcum* have led to fundamental advances in the understanding of the physiology, biochemistry and genetics of N_2_-fixation and H_2_ metabolism. Extensive reviews of aspects of the family *Azotobacteraceae* have been published [13, 14, 15].

Members of the species *A*. *chroococcu*m are common in neutral or alkaline soils whereas *A*. *vinelandii* is rare in soils (summarised in [7]). The many studies of isolates of this species have examined their potential to stimulate plant growth either through the production of plant growth substances, mineralisation or contribution of fixed nitrogen or a combination of these factors. There has long been an interest in their ecological role and in the use of these organisms as agricultural inoculants [16].

*Azotobacter chroococcum* NCIMB 8003 (Ac-8003 hereafter) corresponds to N.R. Smith’s strain No. 7 isolated in the U.S.A. ca. 1934 [17]. It was first reported in a study of the trace element requirements of *Azotobacter* [18]. It was deposited in the American Type Culture Collection (ATCC) in 1950 as strain No. 7 of N.R. Smith or strain X-50 of E.B. Fred (ATCC 4412) and in the UK’s National Collection of Industrial and Marine Bacteria (NCIMB) and catalogued as NCIMB 8003. This strain was one of the 19 *A*. *chroococcum* isolates characterized in detail by Thomson and Skerman [7]. Today, it is probably the best characterised of the many *A*. *chroococcum* strains held by culture collections world-wide. It has been used for the study of molybdenum-containing nitrogenase [19,20, 21], the vanadium-containing nitrogenase [22, 23], the physiological mechanisms protecting nitrogenase from O_2_ damage *in vivo* [24, 25, 26, 27, 28], hydrogenase and hydrogen-recycling [29,30, 31, 32], nitrate and nitrite reductases [33, 34, 35], glutamine synthetase [36], poly-β-hydroxybutyrate synthesis [37] alginate lyase [38] and bacteriophage [39].

The complexity of the genome of a laboratory-habituated and non-gummy derivative of Ac-8003 was described earlier. It was found to contain 6 plasmids pAcX50a, b, c, d, e and f the sizes of which were estimated to be 10.6, 15.2, 63, 71, 136 and 287 Kb respectively [40]. Here we report on the DNA sequence of this organism and compare some aspects of it with that of *Azotobacter vinelandii* DJ (ATCC BAA-1303: Av-DJ hereafter) which was published earlier [41]. Av-DJ is a high frequency transformation derivative of a non-gummy strain CA (OP) (ATCC 13705) the genome sequence of which was published more recently together with that of its tungsten-resistant derivative CA6 [42].

## Results and Discussion

### Summary of the genome

The genome of Ac-8003 comprises 7 replicons totalling 5,192,291bp (Table 1). It contains a circular chromosome of 4,591,803 bp and six circular plasmids pAcX50a, b, c, d, e, f of 10,435 bp, 13,852bp, 62,783, 69,713, 132,724, and 311,724 bp respectively. The genome has an overall G+C% content of 65.69%. The chromosome has a G+C content of 66.27% and the six plasmids are each significantly less G+C rich with contents of 58.12, 55.34, 56.76, 59.23, 61.88, 62.67% G+C respectively.

**Table 1:** Ac-8003 and Av-DJ Genomes Compared.

Organism/Replicon type	Size (bp)	GC%	Protein genes	rRNA genes	tRNA genes	Non-coding RNAs	Pseudo-genes	Total Functional Genes
Ac-8003								
Chromosome	4,591,803	66.3	3,959	18	66	44	72	4,087
Plasmid pAcX50f	311,724	62.7	292	0	0	4	13	296
Plasmid pAcX50e	132,372	61.9	111	0	0	4	10	115
Plasmid pAcX50d	69,317	59.2	55	0	0	3	7	58
Plasmid pAcX50c	62,783	56.7	49	0	0	1	2	50
Plasmid pAcX50b	13,852	55.3	10	0	0	2	0	12
Plasmid pAcX50a	10,435	58.1	9	0	0	1	0	10
Ac-8003 (Totals)	5,192,291	65.7	4,485	18	66	59	104	4,628
Av-DJ								
Chromosome	5,365,318	65.7	4,660	18	64	47	64	4,789

The genome encodes 4628 predicted protein-encoding genes of which 541 (12.2%) are plasmid borne. 3048 (65%) of these show > 85% identity to the 5050 genes identified in Av-DJ and of these 99 are located on the plasmids. 1071 (23%) of the potential protein encoding genes show the highest identity to various species of the genus *Pseudomonas*, 43 to species in the genus *Burkholderia* and smaller numbers to members of the genera *Halomonas*, *Escherichia*, *Azoarcus*, *Singulospharea*, *Aromatoleum*, *Ralstonia*, *Cuprividus*, *Polaromas* and *Acidovorax*. 98 potential protein-encoding genes showed no significant identity to any others in the database. The chromosome contains 6 complete rRNA operons as in Av-DJ and 66 tRNA genes, two more than Av-DJ, one of which is located within a gene cluster encoding a prophage. A comparison of the two genomes is given in Table 1.

Analysis of the methylation status of the genome revealed a complex pattern of 5 methylation motifs of which 4 are asymmetric ^m6^A modifications probably involving Type I DNA methylases. The fifth is a symmetric ^m4^C modification probably involving a Type II or III DNA methylase (Table 2). The methylation pattern for Av-DJ is not yet available for comparison but based on a search for genes encoding restriction modification systems (RMS) it is likely to be less complex.

**Table 2:** The Methylome of *Azotobacter chroococcum* NCIMB 8003.

Methyltransferase Specificity	Modification Type	No. in Genome	No. detected in genome	% detected
5’-GACN_5_CTTC-3’ 3’-CTGN_5_GAAG-5’	^m6^A ^m6^A	1411 1411	1410 1408	99.93 99.79
5’-CCAGN_5_GTG-3’ 3’-GGTCN_5_CAC-5’	^m6^A ^m6^A	698 698	697 697	99.86 99.86
5’-GAGWN_5_GCTC-3’	^m6^A	153	152	99.35
5’-GAGSN_6_CTC-3’	^m6^A	1120	940	84.11
5’-CCTCGAGGA-3’ 3’-GGAGCTCCW-5’	^m4^C ^m4^C	270 389	202 265	74.81 68.12

^m6^A = *N* ^6^-methyladenine, ^m4^C = *N* ^*4*^-methylcytosine; W = A or T, S = C or G

#### Synteny between the Ac-8003 and Av-DJ chromosomes

**Fig 1:**
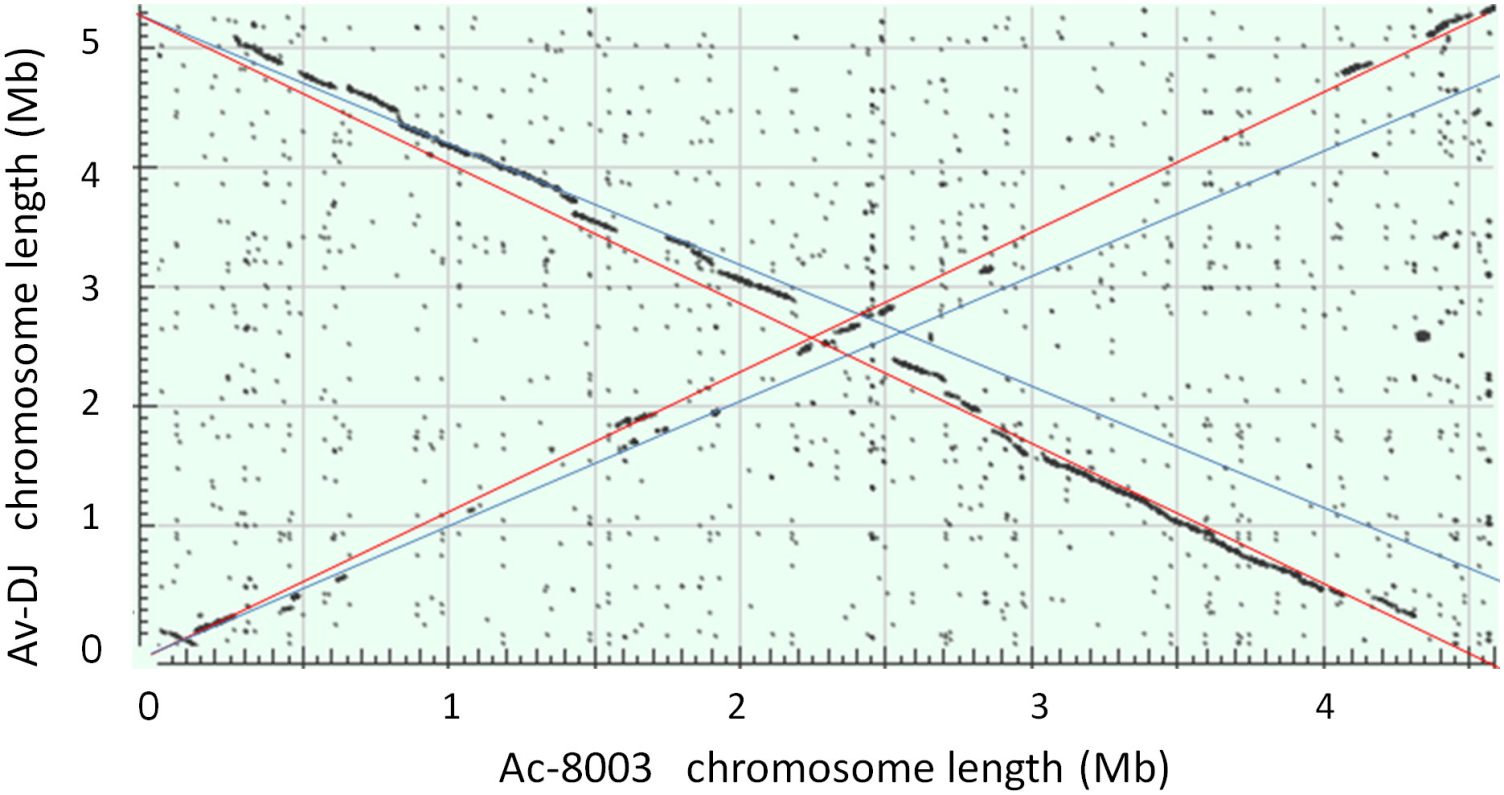
Synteny between the chromosomes of *A*. *chroococcum* NCIMB 8003 (ordinate) and *A*.*vinelandii* DJ (ATCC BAA-1303). The nucleotide sequences of the chromosomes of *A*. *chroococcum* NCIMB 8003 (ordinate) and *A*.*vinelandii* DJ (ATCC BAA-1303) (abscissa) were aligned using BLASTn. The red and blue diagonal lines show the slopes that would be obtained were each chromosome aligned against itself: red, *A*. *chroococcum*; blue, *A*.*vinelandii*.

**Fig 2:**
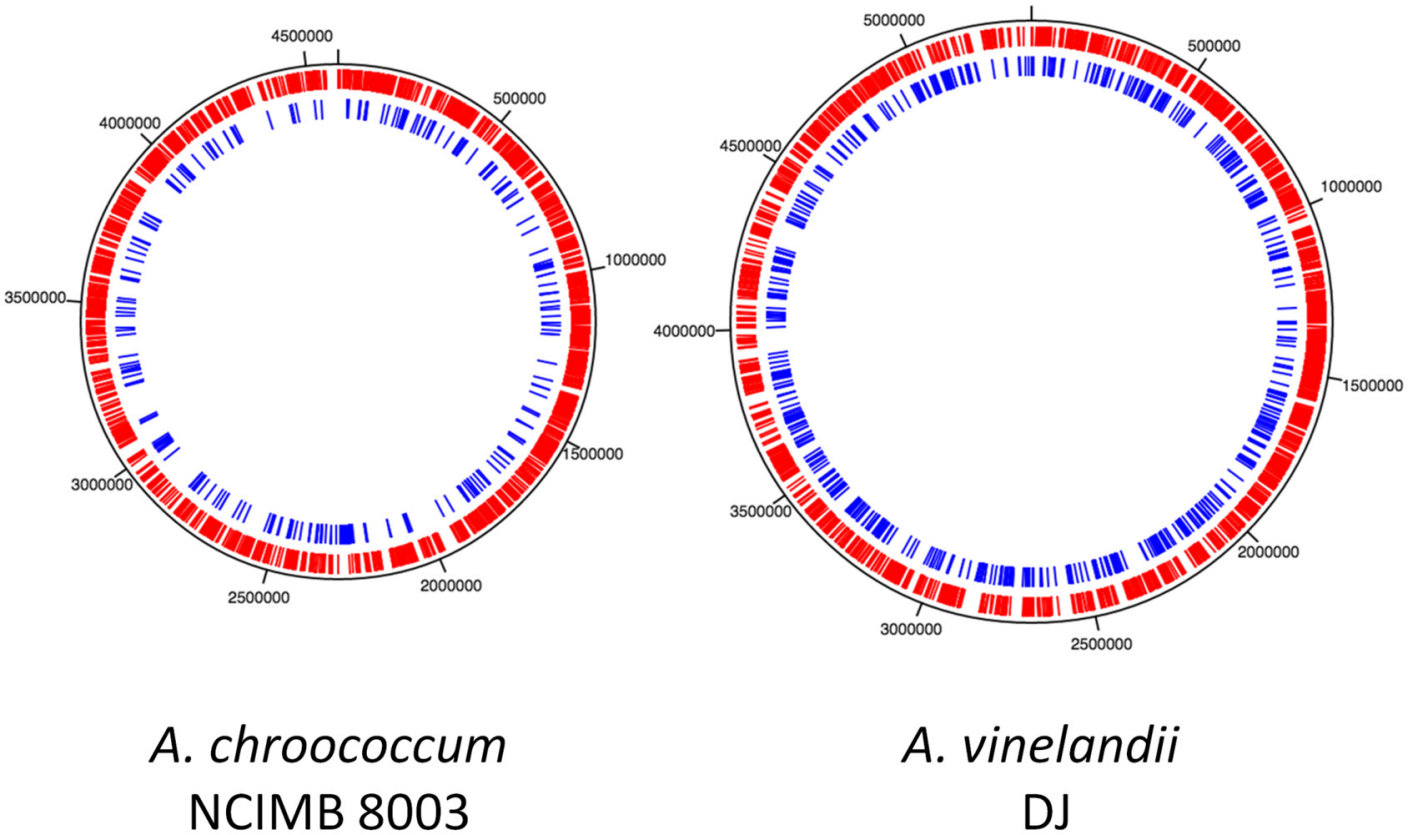
Comparison of the gene contents in the chromosomes of *A*. *chroococcum* NCIMB 8003 and *A*.*vinelandii* DJ (ATCC BAA-1303). Genes present in both strains are shown in red (“core genes”) whilst the genes present in only that strain (“accessory genes”) are shown in blue. The genome dimensions are marked in bp.

The Ac-8003 chromosome is 773.5 Kb shorter than that of the Av-DJ. Despite the difference in the chromosome sizes there is significant synteny throughout the chromosomes as seen in a comparative BLAST alignment of the nucleotide sequences of the two chromosomes (Fig 1). A notable property of the alignment is the “X” shape of the plot. This appears to be a characteristic of the genomes of closely related species and has been explained by exchange and inversion of often large segments of the chromosome from one side of the replication fork to the other [43,44]. Also, it has been observed that in general these exchanges occur more frequently close to the replication origin and terminus as is evident in the Ac-8003 vs Av-DJ alignment. In all but one case these translocations do not appear to result in a significant change in the replication order of the genes. The exception is a region of nearly 100 Kb close to the replication origin of both organisms. The alignment of the two chromosomes suggests that the difference in the sizes of the two chromosomes has occurred by deletion or expansion from an ancestral genome both through step change (insertion or deletion of relatively large segments) and also gradual gain or loss of individual or small clusters of genes throughout the genomes of both organisms as shown (Fig 2).

#### Mobile genetic elements

The Ac-8003 and Av-DJ genomes contain distinct profiles of mobile genetic elements (MGEs). These include plasmids, insertion sequences, transposons, integrating conjugative elements, prophage, introns and inteins. Though a few appear to have been acquired by vertical transmission most appear to have been acquired through horizontal gene transfer (HGT). The Ac-8003 plasmids will be discussed in detail later.

#### Insertion sequences

A striking property of both genomes and especially that of Ac-8003 is the very high content of insertion sequences (IS). The Ac-8003 genome contains 258 transposase (Tn) and potentially inactivated transposase sequences (pseudogenes) (iTns). Pfam analysis shows that the DDE and DUF772 transposase protein families are, at 109 and 65 respectively, the two most frequently occurring protein families in the Ac-8003 genome whereas, in general, ABC transporters or regulatory helix-turn-helix family proteins are most common in free-living bacteria. These Tns and iTns belong to as many as 12 of the 17 recognised Tn families [45] (http://www-is.biotoul.fr/). 177 of these elements are located in the chromosome (~ 1 per every ~26 Kb) whilst 34, 28, 14, 2, 2 are present in plasmids pAcX50f, e, d, c and, b respectively. The most frequently occurring IS (ISAzch1) (e.g. Achr_620) is a member of the IS4 family and encodes a Tn of 369 aas. It occurs 62 times in the total genome, 56 times in the chromosome and 5, 4 and 2 times in plasmids pAcX50f, e, and b respectively. The ISAzch1 aa sequences show > 99% identity which suggests that this IS has been acquired recently and undergone a recent “explosive” expansion throughout the Ac-8003 genome. Orthologs with 89% identity are present in the genomes of several strains of *Pseudomonas aeruginosa* but are absent in Av-DJ.

Av-DJ also has a relatively high number of IS with 137 Tns or iTns from 11 families, several of which have high degrees of identity to Ac-8003 orthologs. The abundance of ISs in these 2 Azotobacter species is high compared to other members of the order *Pseudomonadales*. *Pseudomonas* species average 37 ISs/Tns per strain across 23 strains drawn from 8 species. Abundances range from as few as 3 in *P*. *fluorescens* Pf-5 to as many as 126 in *P*. *putida*.

#### Prophages

The chromosomes of both Ac-8003 and Av-DJ carry large clusters of genes likely to be prophages. In Ac-8003 the cluster spans 33.4 Kb and contains ~32 genes (Achr_20180 to 20550) and in Av-DJ the cluster spans 35.5 Kb and contains 29 genes (Avin_37640 to 37120). There is no significant nucleotide sequence identity between these two putative prophages though over 6 Kb of the Ac-8003 region show good alignment and 72% identity to the genome of *Pseudomonas knackmussii* B13. Regions of 2.5 and 5.6 kb of the putative Av-DJ prophage show respectively 72 and 75% nucleotide sequence identity to *Pseudomonas* phage phi CTX which has a genome of 35,580 bp [46] which is very close to the estimated size of the prophage inserted in the Av-DJ chromosome.

#### Integrating conjugative elements (ICEs)

Whilst Av-DJ does not carry any conjugative plasmids it does carry two large clusters of conjugation genes located relatively close to each other in the chromosome. These contain all the functional elements which characterise mobile genetic elements called Integrating Conjugative Elements (ICEs). The larger of the two clusters (we call ICE-AvDJ1) shows great similarity to the Tn4371 (CMCI-3) ICE family [47]. It extends for 46.3 Kb from a phage integrase family gene (Avin_36120) to a gene encoding a TrbI conjugation protein (Avin_35620). The region is flanked by a number of genes for Tns or iTns and at the distal end contains *tra*RFG and *trb*BECDEJLFGI gene clusters potentially encoding a Type IV secretion system. ICE-like elements can carry a variety of accessory genes e.g. antibiotic resistance genes and multidrug resistance pumps. In Av-DJ, the payload appears to include three Major Facilitator Superfamily (MFS) transporters and adjacent regulatory genes, two adjacent genes potentially encoding a major royal jelly-like protein (Avin_35790) and a ketosteroid isomerase (Avin_35800). The smaller cluster of 34.6 Kb (we call ICE-AvDJ2) appears to be a member of the PFL ICE family and extends from a gene encoding a candidate type III Hop effector protein (Avin_36200) to a gene for a phage integrase-like protein (Avin_36590). ICE-AvDJ2 appears to contain relatively few accessory genes several of which encode conserved predicted proteins. There appear to be no obvious ICE-like elements in Ac-8003 but see the section on plasmids below.

#### Introns, retrons and inteins

The Ac-8003 chromosome contains two type II introns which lie 11 kb apart and encode different reverse transcriptases (Achr_29160, 29060). The first of these shows a high a degree of sequence identity to a type II intron in Av-DJ (Avin_13490) and lies close to the 3’ terminus of the *gro*EL genes of both organisms (Achr_29170; Avin_13490). This intron in *A*. *vinelandii* was observed previously and belongs to a monophyletic subset of bacterial group II introns that share a large insertion at their 5′ extremity [48]. An ortholog appears to be conserved in Ac-8003 though it is not located exactly at the translation termination codon of *gro*EL. Av-DJ contains a further 5 introns (Avin_18750, 24600, 24740, 45820, 27540) and two potential retrons (Avin_344406, 345515).

The chromosome of Ac-8003 contains two inteins both of which are located in the genes encoding the large subunits of the ribonucleotide reductases (NRDs). The gene product of the *nrd*A gene (Achr_15980) encoding the α-subunit of the Type I NRD contains an intein (aas 442 to 800) which is conserved in the Av-DJ ortholog (Avin_33290). The second intein occurs between aas 321–690 in the gene product of *nrd*Ja (Achr_580) for the type II B12-dependent NRD in *Ac*-8003. This second intein is absent in the Av-DJ ortholog (Avin_00810) and also all *Pseudomonas* species in the database but is potentially present in *Rhodanobacter fulvus* and a few γ- and β proteobacteria. Both inteins appear to encode homing endonucleases of the LAGLIDADG-3 family but share weak sequence identity to each other. The presence of inteins in prokaryotes is rare but where they occur they are frequently located in ribonucleoside reductases as first seen in several *Archaea* [49] and the cyanobacterium *Anabaena* 7120 [50].

### Biosynthetic and metabolic activities encoded in the chromosome

#### Carbon and energy substrates

Detailed physiological and numerical taxonomic studies have been carried out on all species in the genus *Azotobacter* drawn from culture collections world-wide or isolated by the authors. The strains studied included 19 isolates of *A*. *chroococcum* including NCIMB 8003 and 17 isolates of *A*. *vinelandii* including O and OP the progenitor of Av-DJ [7]. Carbon utilization patterns formed a significant part of this numerical taxonomic study. The physiological differences observed between the members of these two species are strikingly well reflected in the gene complements of the two examples sequenced to date. The genome analysis also reveals the potential for these two Azotobacters to use a number of other substrates not previously tested.

#### Mono-, di- and trisaccharides

Thompson and Skerman [7] showed that Azotobacters can generally use the following as sole C sources: mono-, di- and tri-saccharides, primary and sugar alchohols, short-chain organic acids, medium chain length fatty acids and aromatic compounds with some slight variations in the detailed substrate range. Both *A*. *chroococcum* and A. *vinelandii* use the hexoses; fructose, galactose, and glucose. However, *A*. *vinelandii* strains also use rhamnose and Av-DJ contains genes potentially encoding an L-rhamnose mutarotase and rhamnose-proton symporter (RhaUT; Avin_09140, 09150) which are absent from the *A*. *chroococcum* genome. The use of trehalose as a carbon and energy source was shown to be common in *A*. *chroococcum* strains including Ac-8003 (also *A*. *nigricans and A*. *armeniacus*) but rare in *A*. *vinelandii* [7]. This difference appears to be explained by the presence in the chromosome of a potential trehalase-encoding gene (*tre*A) in Ac-8003 (Achr_32920) which is absent from Av-DJ. The TreA protein has a potential-terminal signal sequence for its export at least to the periplasm and shows over 70% identity to orthologs from *Pseudomonas Sp*.HPB0071 and *Xanthomonas translucens*. Both species generally use the trisaccharides melezitose and raffinose.

#### Polysaccharides (starch, glycogen, levan, agar)

A distinctive feature of almost all *A*. *chroococcum* strains and a subset of strains of *A*. *armeniacus* but which is not seen in *A*. *vinelandii* or other Azotobacters is their ability to hydrolyze starch. Also, Ac-8003 is one of a limited number of *A*. *chroococcum* strains that can also hydrolyze glycogen. Starch and glycogen breakdown require the excretion of both polyglucan α-1,4 and α-1,6 glucosidases (α-amylases amylases and debranching enzymes). Ac-8003 contains two chromosomal genes apparently encoding α-1,4 glucosidases (Achr_21940, 35970) which show 76% identity to each other and 89% and 75% identity respectively to an ortholog in Av-DJ (Avin_26980). Both the Achr_21940 and the Av-DJ ortholog (but not Achr_35970) are predicted to contain N-terminal signal export sequences. Ac-8003 contains three genes encoding alpha-1,6 glucosidases two of which are not found in Av-DJ and which encode polypeptides with N-terminal export signal sequences. One (Achr_35980) is chromosomal and the other is located on plasmid pAcX50e (pAcX50_940). Their gene products show respectively 94% and 53% identities to orthologs in *Cellvibrio sp*. *BR*. Achr_35980 lies adjacent to, but is divergently transcribed from, the gene encoding one of the Achr_35970-encoded glucan alpha-1,4 glucosidases described above. Approximately 3 Kb upstream of the chromosomal debranching enzyme gene is a large operon comprising 9 *gsp* genes (*gsp*D,E,F,G,H,I,J,K,L: Achr_361010 to 36100) potentially encoding a general secretion pathway for protein secretion across the outer membrane. This pathway may be involved in the export of both the polyglucan α-1,4 and α-1,6 glucosidases in Ac-8003. Though Av-DJ can potentially secrete an α-1,6 glucosidase, the reason why it may not be able to use starch and glycogen as a C-source is that it cannot secrete its glucan α-1,4 glucosidase.

A highly distinctive feature of *A*. *chroococcum* strains is their ability to produce an agar-diffusible homopolysaccharide when grown on sucrose or raffinose. This is evident as a “cloudy” ring around young colonies on agar plates but which fades in time. It is likely that this is a levan and the genome of Ac-8003 contains a gene (*sacB*) encoding a levansucrase (Achr_16320) with an N-terminal signal sequence with 82% identity to genes from *Vibrio sp*. *EJY3*. This gene is separated only by an IS6 transposase gene from a gene (*sacC*) encoding an endolevanase (Achr_16340) that lacks a signal sequence and also shows the closest match to *Vibrio sp*. *EJY3*. This pair of functionally related genes is often seen in other bacteria and in Ac-8003 they appear to be a potentially mobile functional unit for levan formation and breakdown. Ac-8003 contains a second *sac*C gene elsewhere in the genome encoding an endolevanase (Achr_26340) with a high degree of identity to orthologs in *Streptomyces* sp. but which does contain an N-terminal signal peptide. It is possible that the levan enzymes are exported via the *gsp*-encoded general secretion pathway discussed earlier.

An interesting observation is that both Ac-8003 and Av-DJ can potentially secrete a β-agarase (agarose 3-glycanohydrolase) which are the products of putative *aga*A genes in the two strains (Achr_15560: Avin_19460). Both genes encode proteins with potential N-terminal signal sequences and show the highest identity (~60%) to gene products from a wide variety of *Pseudomonas* sp. including *P*. *alcaligenes*, *P*. *chloraphis and P*. *fulva*. Under what conditions this gene might be expressed is unknown. To our knowledge, breakdown of agar has not been observed in growth of either Ac-8003 or Av-DJ on solid media, though when grown on nutrient agar we have observed that colonies of Ac-8003 appear tenaciously adhered to the agar.

#### Alcohols and polyols

*A*. *chroococcum* and *A*. *vinelandii* strains can use the primary alchohols ethanol, butan-1-ol and pentan-1-ol and the sugar alcohols mannitol and sorbitol and myo-inositol. However, unlike *A*. *vinelandii*, no *A*. *chroococcum* strains use glycerol, erythritol and D-arabitol and use of myo-inositol is exceptional.

#### Organic acids

The genome sequence indicates that Av-DJ but not Ac-8003 can take up and oxidize glycolate though the cluster of genes for glycolate/lactate permease (*glc*A/*lct*P: Avin_43070) and glycolate oxidase (*glc*DEFG) Avin_43300 to 43330). Glycolate is a natural product of phytoplankton in aquatic environments and appears to be produced in order to manipulate the natural flora [51]. *A*. *vinelandii* strains can also use tartrate and Av-DJ contains the *ttdA*B operon encoding the two tartate dehydratase subunits (Avin_31190, 31200) absent in Ac-8003. Myo-inositol catabolism in Av-DJ is probably dependent on the *iol*EBTH genes (Avin_50050 to 50070, 50120) and *idh*A (Avin_50110). By contrast, the genome sequence of Ac-8003 shows that it probably metabolizes tricarballylate as a C and energy source through the presence of the *tcu*AB genes encoding tricarballylate dehydrogenase and the tricarballylate utilization protein B (Achr_2780, 2790).

#### Fatty acids

Thomson and Skerman [7]showed that all *A*. *vinelandii* strains tested used both C6 (caproate) and C8 (caprylate) short-chain fatty acids, whilst all *A*. *chroococcum* strains tested used caproate but none used caprylate. Both Ac-8003 and Av-DJ contain genes encoding a short-chain fatty acid transporter (Achr_25980; Avin_33780) though they appear to have different evolutionary origins. Av-DJ also has a gene encoding a long-chain fatty acid transporter (Avin_34900) but this is absent in Ac-8003. The inability of Ac-8003 to use caprylate may be explained by different specificities of the short-chain fatty acid transporters and/or the absence of the long-chain fatty acid transporter gene. The presence of the latter gene suggests that Av-DJ may be able to use fatty acids of longer chain lengths than 8 but this has not been reported to our knowledge. Both organisms have an almost completely conserved 7 or 8 gene fatty acid oxidation cluster (Achr_26420 to 26350; Avin_16380 to 16440, 16700). However, Ac-8003 also contains a second fatty acid oxidation cluster comprising 10 genes (Achr_20990 to 20890) which is absent in Av-DJ. All but one of the gene products show between 67 and 90% identity to orthologs from various *Pseudomonas* species. The exception is Achr_20950 which potentially encodes an electron transfer β-protein with the highest identity (>90%) to *etf*B1 and *etf*2 orthologs in Av-DJ (Avin_1510, 14280). Interestingly, the Ac-8003 gene cluster is enclosed between, on the proximal side, a two gene cluster encoding cointegrate resolution proteins S and T (Achr_21010 and 21000) and, on the distal side, a gene for a site specific /phage integrase family protein (Achr_28980). All three genes show the highest identity to orthologs from Av-DJ. This suggests that Ac-8003 has acquired this fatty acid oxidation gene cluster through HGT.

#### Aromatic compounds

Amongst the genus *Azotobacter*, all strains of *A*. *vinelandii* and a few strains of *Azotobacter beijerinckia* are able to use phenol whilst resorcinol usage is a unique and almost invariant C-source for strains of *A*. *vinelandii*. Av-DJ appears to be able make two multi-component phenol hydroxylases encoded by the *dmp*O,N,M,L,K (Avin_08820 to 08860) and the *lap*O,N,M,K gene clusters (Avin_30720 to 30760). *A*. *chroococcum* strains do not use phenol [7] and do not contain the *dmp* and *lap* gene clusters. Hardisson *et al*. [52] demonstrated that several strains of *A*. *chroococcum*, *A*. *vinelandii*, and *A*. *beijerinckii* used benzoate *via* the meta-cleavage pathway through catechol whilst *p*-hydroxybenzoate was metabolised *via* an ortho-cleavage pathway through protocatechuate. Av-DJ is able to use these aromatic compounds and contains several gene clusters required for the uptake and breakdown of benzoate (*ben*ABCD; *mhp*D, *xyl*JI; *dmp*I, *xyl*EJK) and hydroxybenzoate (*pca*GH; *pca*BCDF; *fad*A). Av-DJ is typical of *A*. *vinelandii* in being able to use phenylpropionate (though not phenylacetate) and contains a cluster of genes likely to encode enzymes required for this pathway (*mhp*B, ORF1,2; *mph*CRDFET). However, Thompson and Skerman [7] reported that Ac-8003 is one of a minority of *A*. *chroococcum* strains unable to use benzoate, hydroxybenzoate or phenylpropionate. They were also unable to find biochemical evidence for meta- or ortho-protocatechuate cleavage pathways. Surprisingly, all the gene clusters for the metabolism of the aromatic compound metabolism described above for Av-DJ appear to be highly conserved in Ac-8003. It is unclear why Ac-8003 is cryptic for the use of aromatic compounds. Possibly it contains one or more mutations in structural genes or possibly regulatory mechanisms. Interestingly, it has been reported that catechol dissimilation was plasmid-borne in one strain of *A*. *chroococcum* [53])

### Core biosynthetic and energetic capacities

The essential biosynthetic capacities, macromolecular and energetic machineries of Ac-8003 and Av-DJ appear to be highly conserved and chromosomally located. The initial description of the genome of Av-DJ included a thorough analysis of the genetic basis of the proposed mechanisms underlying the ability of the Azotobacters to fix N_2_ under aerobic conditions. Gene clusters for three hitherto unsuspected potential O_2_-sensitive enzyme systems were also discovered in Av-DJ: CO-dehydrogenase, formate dehydrogenase and also a soluble hydrogenase complex in addition to the previously characterised membrane-bound NiFe-hydrogenase. The presence of these three gene clusters in both organisms suggest that under some circumstances Azotobacter can generate energy by the oxidation of these C1 compounds potentially coupled to the H_2_ evolution. To our knowledge, this possibility has not previously been explored in physiological or biochemical studies. An interesting major difference is the presence of a single ATP synthase operon in Ac-8003 (Achr_40700 to 40630) as compared to two operons in Av-DJ (Avin-52150 to 52230; Avin_19670 to 19750). The Ac-8003 operon is located very close to the origin of replication and shows the highest identity to the similarly located operon in Av-DJ (Avin_52150 to 52230).

The 6 gene *coo* gene operon for CO-dehydrogenase subunits and accessory proteins (*coo*F,S,ORF,C,T, J) are conserved (Achr_4460 to 4410; Avin_04500 to 04450)). The first five predicted polypeptides are most closely related to orthologs from thermophiles and extreme thermophiles such as *Thermococcus* and *Carboxydothermus hydrogenoformans* but have not yet been identified in any other Proteobacteria. The Ac-8003 genome also contains a putative *coo*A gene for a CO regulator which is also present in Av-DJ (Achr_4630; Avin_47010).

The Ac-8003 genome also contains the putative formate dehydrogenase (*fdhDGHIE*) gene cluster (Achr_37970 to 37930; Avin_03800 to 10840) with similarity to the nitrate-inducible FDH from *Enterobacteria*.

A cluster of 6 genes encoding a potential soluble NAD^+^-reducing hydrogenase was also discovered in the Av-DJ genome (Avin_04360 to 04410). This gene cluster is also conserved in Ac-8003 (Achr_4330 to 4370) and lies almost adjacent to the *coo* gene cluster as in Av-DJ. Setubal *et al*. [41] pointed out the similarity of this gene cluster to the six-gene-encoded soluble H_2_-sensing hydrogenase in *Ralstonia eutropha*. However, the organization of this cluster is not well conserved and there is no evidence for the presence of genes similar to *hox*J and *hox*A which encode a signal transduction complex transmitting the presence of H_2_ detected by the sensor hydrogenase in *Ralstonia*. The *Azotobacter* gene cluster appears better conserved in diverse diazotrophs e.g. *Beijerinkia indica*, *Frankia* sp CcI3, *Cyanothece sp*. *7425* and *Acidithiobacillus ferrooxidans* However, the possibility that this gene cluster encodes a sensing hydrogenase in *Azotobacter* is intriguing. There has long been evidence that H_2_ stimulates the expression of the membrane-bound NiFe-hydrogenase in both Ac-8003 [54] and *A*. *vinelandii CA* [55] though no mechanism has yet been identified by which this occurs. It will be interesting to know under what conditions this potentially “soluble” hydrogenase is expressed in Azotobacter as no other H_2_ oxidizing or evolving activities (apart from nitrogenases) have been detected in numerous studies of H_2_ metabolism in both Ac-8003 and *A*. *vinelandii* including knock-out mutants in the structural genes of the membrane-bound NiFe “uptake” enzyme (*hox*KG*/hup*SL).

#### Respiratory chain components

Azotobacters can achieve unusually high rates of respiration and like many other bacteria have complex branched electron transport chains which enable adaptation to varying ambient O_2_ concentrations. The physiological properties of respiration in Ac-8003 in particular the adaptation to enable N_2_ fixation to function in aerobic environments (the so-called “respiratory protection” hypothesis) appears to be a specialization of Azotobacters and has been studied in detail (reviewed in [56]). However, though not studied to the same extent, on the basis of genome analysis, the detailed biochemistry and genetics of respiration in Ac-8003 compares well with the system in *A*.*vinelandii*.

Ac-8003 mirrors Av-DJ almost completely in relation to energy coupling systems. These systems include: the contiguous 13 *nuo*ABCEFGHIJKLMN gene cluster-encoded proton-translocating NADH-quinone oxidoreductase (Achr_19340 to 19220; Avin_28440 to 28560)(Complex I); the *sdh*CDCB operon and the adjacent *suc*AB, *lpd*A and *suc*C genes for succinate/fumarate reductase (Achr_18040 to 18120; Avin_29810 to 29740) (Complex II); and *pet*A,B,C (Achr_29610 to 29520; Avin_13060 to 13080) encoding the ubiquinol cytochrome c reductase (Complex III). In Ac-8003 PetA which encodes the Rieske-like Fe-S subunit (Achr_29610) has a longer C—terminus which shows higher overall identity to comparable length genes in several Pseudomonads rather than in Av-DJ in which the ortholog is Avin_13060. Both Azotobacters contain highly conserved *hup/hox* gene clusters encoding membrane-bound H_2_-oxidation-cytochrome *b* enzyme:ubiquinone oxidoreductase complex and accessory genes (Achr_39120 to 39210; Avin_50500 to 50590) and the adjacent contiguous hypABFCDE cluster of genes for Ni assimilation and NiFe cluster formation. (Achr_39110 to 39060; Avin_50490 to 50440).

Both organisms have the genetic capacity to synthesize as many as 5 terminal oxidases which was unexpected from biochemical studies. Both genomes carry genes for three likely heme-copper cytochrome/quinol terminal oxidase systems (referred to here for Ac-8003 as CytOx-1, 2, 3). These comprise the contiguous *cco*NOQPGHIJ gene cluster (Achr_24920 to 24990; Avin_20010 to 19940) for a potential proton-pumping *cbb*3-type cytochrome oxidase (CytOx-1). This is located immediately upstream of related biogenesis/maturation genes, a *cyc*Z-ortholog (Achr_25000; Avin_19930) and *hem*A (Achr_25010; Avin_19920). CytOx-2 is encoded by a 10 gene cluster (Achr_390 to 480; Avin_01020 to 00930) which contains the three structural genes for subunits I, II and III and also a cluster of biogenesis/maturation genes. CytOx-3 appears to be encoded by a cluster of 6 genes (Achr_30990 to 31050; Avin_11200, 11180, 11170) with structural genes for subunits I and II but apparently no subunit III. Subunits I of CytOx-2 and -3 show 43% identity over the N-terminal ~ 540 aas with each predicted to contain 12 membrane spanning helices. However, subunit I of CytOx-3 is 290 residues longer than CytOx-2 equivalent and this C-terminal domain resembles subunit III of CytOx-2 in containing 7 membrane-spanning helices but with no strong overall sequence identity. This suggests that subunits I and III are fused in CytOx-3. Also, subunit III shows identity to major facilitator superfamily transport proteins which suggest that the reduction of O_2_ by these enzymes may be coupled to solute transport rather than contributing directly to the H^+^ gradient.

There have been many biochemical and genetic studies of the quinol-oxidising cytochrome *bd* encoded by the *cydAB* genes in *A*. *vinelandii* and especially of their role in respiratory protection of nitrogenase [56]. Surprisingly, genome analysis revealed that the organism has a second set of *cydAB* genes which are also highly conserved in *A*. *chroococcum*. CydAB1 in Ac-8003 shows high sequence identity to the well-studied system in Av-DJ and likewise is located in a 1.4 Kb region 3’ to the *cco* gene cluster in which is located the *cyd*R gene (Achr_25020; Avin_19910) found to be a negative regulator of CydAB in *A*. *vinelandii* [57]. The Ac-8003 cluster comprises 5 genes, four of which are *cyd*A1, *cyd*B1, *cyd*X (*ybg*T), *ybg*E (Achr_25030 to 25060; Avin_19890 to 19860) as in Av-DJ. CydX has recently been shown to be a third subunit of the membrane-bound enzyme in *E*. *coli* [58, 59]. This gene potentially encodes a polypeptide of 71 aas predicted to contain a central single membrane-spanning helix. CydB1 belongs to the family of these proteins characterized by having a long hydrophilic Q (quinone-binding) loop between membrane helices 6 and 7 [60]. There appears to be a further potential gene at the 5’ end of the operon which is not present in Av-DJ but is found in *Pseudomonas pseudoalcaligenes* (64% identity) and a number of other γ-proteobacteria.

In Ac-8003 CydBD2 is encoded by the co-transcribed *cyd*A2B2 genes (Achr_31130, 31140: Avin_11050, 11040) situated 11.7 kb from the CytOx-3 gene cluster described above. These genes do not appear to be co-transcribed with any other genes. CydB2 and CydD2 both show 92% identity to their equivalents in Av-DJ but only 30 and 31% identities respectively to Ac-8003 CydA1 CydB1. In contrast to CydAB1, this enzyme belongs to the second family of these enzymes characterized by a short hydrophilic Q loop between membrane helices 6 and 7 [60].

Another component of the respiratory protection system in *A*. *vinelandii* was the identification of the FAD-dependent, uncoupled so called “respiratory” NADH:ubiquinone oxidoreductase [61,62] encoded by the monocistronic *ndh* gene in Av-DJ (Avin_1200). This gene is also highly conserved in Ac-8003 (Achr_30570).

### Ion Pumps, porters and channels

Thompson and Skerman [7] showed that *A*. *chroococcum* and *A*. *vinelandii* strains are moderately alkaliphilic growing across the pH range from 6.5 to at least 10. They also reported that they were tolerant to 0.175M NaCl but not 0.35M. However, Madkour *et al*. [63] showed that a number of strains of *A*. *chroococcum* could tolerate at least 0.5M NaCl. Interestingly, Na^+^-dependent-growth has been described for several strains including ATCC 7493 and the type strain ATCC 9043 [64]. However, it is not known if Ac-8003 has a similar sodium dependency. Madkour *et al*. [63] studied the adaptation of *A*. *chroocooccum* to osmotic stress in a laboratory strain and two strains isolated from the rhizospheres of salt-tolerant plants and showed that different osmolytes accumulated under osmotic stress. Glutamate and proline levels were elevated in young cultures but these appear to be replaced by trehalose when organisms are grown for longer periods.

#### K^+^ uptake/efflux

Ac-8003 and Av-DJ have several conserved chromosomally-encoded membrane bound systems which potentially could play a part in maintaining/regulating K^+^ levels. However, Ac-8003 has at least three additional types of systems. Systems conserved in the two bacteria are: the low affinity K^+^ uptake protein orthologous to TrkH (Achr_27210; Avin_15550), and TrkA (Achr_1110; Avin_00140) though we were unable to identify an ortholog to TrkG which is a regulator of TrkH in *E*. *coli*. Also identified were KupA (Achr_19820; Avin_24540) which is the distal cistron co-transcribed with KdpD1 and KdpE1 (Achr_19800, 19810; Avin_24520, 24530) which are the regulators of KupA and a second pair of *kdp*DE-like genes (*kdp*D2,E2: Achr_31570, 31580: Avin_10590, 141251). The genomes of both organisms contain genes potentially encoding two K^+^/H^+^ antiporters: a CPA2 Kef-type family (Achr_31660; Avin_10490) and NhaP2/cell volume regulator (Achr_6800; Avin_45090).

Ac-8003 appears to have three interesting types of systems not found in Av-DJ. The first is a potential high-affinity K^+^-transporting ATPase encoded by a 7 gene operon comprising the structural genes *kdp*A’,A2”,B,C,F (Achr_11210 to 11170) and two adjacent genes which appear to encode an KdpD-like osmosensitive K+ channel signal transduction histidine kinase and KdpE-like cognate signal regulator (Achr_11160, 11150). Proteins encoded in this cluster show the highest sequence identity to orthologs from the β-proteobacterium *Dechloromonas aromatica* RCB. The second functional difference is the presence of three additional K^+^ channels. One apparently encodes a K^+^ channel (Achr_40040) similar to orthologs found in Pseudomonads. The other two appear to encode different voltage-gated K^+^ channels, one belonging to the KQT family (Achr_25330) with 76% identity to an ortholog from *Pseudomonas stutzeri* and the other (Achr_2930) to the Kch family showing 44% identity to an ortholog from *Cupriavidus* sp. HMR-1. The third major difference is that Ac-8003 contains a KefB-like glutathione-regulated K^+^-efflux system (Achr_20870). This gene appears to be co-transcribed with a gene (*gdh*) encoding a glutamate synthase (Achr_208803). There is a second glutamate synthetase gene in the chromosome of Ac-8003 (Achr_3890) however this shows 87% identity to an ortholog in Av-DJ (Avin_25670). These findings suggest that it may be of selective value to Ac-8003 to acquire K^+^ from a low K^+^ environment and that the presence of a number of voltage-gated K^+^ channels and a glutathione-regulated K^+^ efflux suggest that it may be subject to greater osmotic and electrophile challenges than Av-DJ.

#### Na^+^ channels/pumps and porters

There is strong evidence that Azotobacters employ both H^+^ and Na^+^ motive force (NaMF). Both strains have a number of chromosomal gene clusters potentially contributing to Na^+^ efflux and a number of systems which use Na^+^ to support solute uptake. These include: the Nqr—like Na^+^-translocating NADH-quinone reductase encoded by the *nqr*ABCDEF operon (Achr_28250 to 28200: Avin_14590 to 14640); two putative redox-driven monovalent (Na^+^) ion pumps encoded by the pair of *rnf* operons reviewed by Biegel *et al*. [65], *rnf*A1,B1,C1,D1,G1,E1,H1 (Achr_39410 to 39350: Avin_50980 to 50920) and *rnf*A2,B2,C2,D2,G2,E2 (Achr_15320 to 15180: Avin_ 19270 to 19220). The first *rnf* cluster is linked to the minor *nif* cluster. Both organisms contain an Na^+^/H^+^ NhaP2-family antiporter or cell volume regulation protein A (Achr_16790; Avin_31800). Ac-8003 contains two *mnh*(*sha*,*pha*) A/BCDEFG genes encoding potential NADH:ubiquinone oxidoreductase monovalent cation/Na^+^ antiporters (Achr_17400 to 17450; Achr_15550 to 15500). Polypeptides encoded in the first of these show high sequence identities to Av-DJ orthologs (Avin_19530 to 19580). However, the second system is apparently not present in Av-DJ but but shows between 56 and 77% identities to orthologs in several *P*. *mendocina* strains. Both Ac-8003 and Av-DJ contain two additional Na^+^/H^+^ antiporters (Achr_11320; Avin_39030: Achr_40120; Avin_51740).

#### Osmotic control

Osmotic control in Azotobacter may be of particular interest given the ability of Azotobacters to form dessication-resistant cysts. Ac-8003 and Av-DJ appear to contain two and three mechanosensitive conductance channels respectively. The two common channels show high sequence identity to each other and comprise the small conductance channel MscS encoded by *msc*S (Achr_6790: Avin_45100) which is co-transcribed with the gene for the cell volume regulation protein A (described above) and the large conductance channel MscL encoded by mscL (Achr_9360: Avin_41090). A gene encoding a second small mechanosensitive conductance channel in Av-DJ (Avin_20120) is absent from Ac-8003.

### N_2_-fixation

The genome sequence confirms earlier findings that Ac-8003 contains the molybdenum and vanadium nitrogenase systems but none of the genes which are specific for the third *anf*-encoded Fe-only system. The genes for the molybdenum system (*nif*) and the vanadium system (vnf) genes are located in several locations in the genome. The largest cluster of *nif* genes is almost identical in composition and arrangement to that described in *A*. *vinelandii* (Avin_01380 to 0710). It comprises 31 contiguous genes (Achr_1260 to 1570) arranged unidirectionally, with one exception, into potentially 8 operons. It begins with the structural genes *nif*HDK for the Fe-protein and MoFe-protein followed by genes involved in assembly and insertion of the metal cofactor clusters including the FeMo-cofactor (FeMo-co) and the potential electron donor to the enzyme, flavodoxin. One difference in the clusters is the presence of a gene for a 4Fe-4S ferredoxin of 97 aas (Achr_1380). This lies immediately upstream of, and appears to be co-transcribed with, the gene (Achr_1390) encoding the equivalent of the 2Fe-2S Shethna I protein (Avin_01520). This gene is not present in Av-DJ and the best match shows 96% identity to an ortholog in *P*. *stutzeri* which is also located upstream of a small 4Fe-4S ferredoxin within the major *nif* cluster in that organism.

The gene encoding the 2Fe-2S ferredoxin (*Ac*P) which forms the tripartite complex associated with conformational protection from O_2_ damage of the Mo-nitrogenase *in vivo* in Ac-8003 [27] is not included in the major *nif* cluster. The N-terminal amino acid sequence of *Ac*P that we determined earlier shows it to be Achr_10540 which corresponds to the Shethna II protein (Avin_39700) which also protects nitrogenase from O_2_ in *A*. *vinelandii* [66, 67]. Given that it can be co-purified as part of the MoFe-nitrogenase complex, it is surprising that the gene for this protein is encoded on a monocistronic operon and is not part of any *nif* or *vnf* gene clusters. Conformational protection is characterised by a switch off/switch on of nitrogenase activity when N_2_-fixing organisms are exposed to a raising and lowering of oxygenation [26]. The switch off/switch on phenomenon is also observed for the V-nitrogenase (R. Robson, unpublished work) so it is possible that *Ac*P may also protect the V-nitrogenase from O_2_ damage *in vivo*.

A second cluster of *nif* and potentially functionally-related genes is located elsewhere in the genome and is also highly conserved in composition and organisation as in the minor *nif* region in *A*. *vinelandii* which appears to comprise 8 genes (Avin_50990 to 51060). This cluster comprises one operon for the two component regulator system *nif*A L (Achr_39420, 39430) and a second operon comprising 6 genes (Achr_39440 to 39490) starting with *nif*B and including *nifQ* required for synthesis of the FeMo-cofactor. In both organisms this gene cluster is followed by the *rnf*A1 *to* H1 gene cluster encoding one of the two potential Na^+^-dependent NADH-ubiquinone reductases described above.

In Av-DJ the *rnf*A1 operon is followed by a cluster of 15 genes probably constituting a genome island for a potential Type IV secretion system (Avin_50750 to 50890) which were first identified in *Vibrio cholerae*, *Burkholderia mallei and Pseudomonas aeruginosa* [68]. Ac-8003 does not appear to contain an equivalent system.

In Ac-8003 and Av-DJ the *rnfA1 to H1* operon also appears to have a further two genes potentially encoding a putative Fe-Mo-co biosynthesis protein NafY (Achr_ 39340: Avin_50910) and a further nitrogen-fixation related protein (Achr_39330, Avin_50900). This operon is followed by eleven genes potentially arranged into 3/4 operons which appear to be involved in the acquisition of molybdenum including an ABC-type transporter including a TonB receptor potentially for molybdate (Achr_39320 to 39290), *mod*G (Achr_ 39280, 39270), two divergently transcribed *mod*E/G genes for Mo-processing homeostasis and a further ABC transporter encoded by the *mod*A1B1C1 genes (Achr_39260 to 39230). In Av-DJ, this cluster (Avin_50730 to 50650) is almost entirely conserved except that it lacks the TonB receptor gene present in the Ac-8003 cluster (Achr_39300).

The *nif*AL operon in Ac-8003 lies at one end of a large assemblage of genes involved in Fe and Mo acquisition, metalloenzyme synthesis and maturation. This includes genes for the membrane-bound NiFe-hydrogenase and Ni assimilation/cofactor processing described earlier (Achr_39210 to 39060), and a cluster of 4 potentially monocistronic genes for membrane proteins including a potential Ni permease (Achr_39050 to 39020). It also includes a large cluster of 15 genes potentially for Fe uptake and the synthesis and export of a non-ribosomal peptide siderophore (Achr_39010 to 38860) discussed further below. The comparable location in Av-DJ contains a 3 gene operon potentially encoding an ABC-type ferrichrome transporter (Avin_39050 to 39030) and is followed by genes not involved in metal assimilation or metallo proteins. In Ac-8003, the assemblage terminates with the *vnf*UAENX cluster (Achr_38740 to 38780) for the regulation and maturation of the V-nitrogenase. The structural genes for the V-nitrogenase are clustered elsewhere in the genome and close to the major *nif* cluster.

The V-nitrogenase structural gene cluster was reported previously and comprises two operons one encoding the *vnf*H-for the Fe-protein and a gene for a ferredoxin and potential electron donor to VnfH (Achr_2560, 2550) and the other the *vnf*DGK genes for the VFe-protein (Achr_2530 to 2510: Avin_02610 to 02590). This study confirms that in Ac-8003, these two operons are separated by a monocistronic operon containing a gene (Achr_2540) absent in the equivalent region in Av-DJ. Disruption of this gene did not obviously affect V-dependent N_2_-fixation in Ac8003 [69] and growth more generally. Interestingly, BLAST analysis shows a second copy of this gene in Ac-8003 (Achr_21990) whose gene product shows 77% identity to that of Achr_2540. Orthologs exist in a number of other organisms, the closest matches at the protein level being to gene products in *Aromatoleum aromaticum* (76%) and *Ectothiorhodospira* sp. PHS-1 (72%). It is notable that several orthologs of this gene are present in these organisms and especially in the genome of the alkane degrading and denitrifying γ-proteobacterium HdN-1. The function of this gene is unknown but domain analysis suggests that it may be an AAA-ATPase with altronate and galactorate dehydratase/hydrolase activity. In both organisms the *vnfDG*K operons are followed by a group of 6 genes (Achr_2500 to 2450; Avin_02580 to 02530) encoding a conserved uncharacterized protein, VnfX, and a potential phosphonate/phosphite ABC transporter. Given that phosphonates are competitive inhibitors of vanadate it is possible that this transporter may be involved in vanadate uptake.

*Setubal et al*. [41] found two genes encoding proteins (Avin_33440, Avin_47100 denoted as VnfA2 and VnfA3 respectively) which show significant identity to NifA, VnfA and AnfA involved in the regulation of the three nitrogenase systems in Av-DJ. Though the significance of these additional genes is not yet known, it is interesting to note that the Achr_4550 gene product shows 92% identity to VnfA3 (and only 64% identity to VnfA) but VnfA2 appears to be absent.

Earlier studies showed that nitrogenase activity *in vivo* in *A*. *chroococcum* NCIMB8003 was inhibited when organisms were subjected to an ammonium shock. The inhibition was the result of post-translational modification of the Mo-nitrogenase Fe protein probably involving ADP-ribosylation [36]. We have observed that the V-nitrogenase in *A*. *chroococcum* is also reversibly inhibited by ammonium shock (unpublished data). Regulation of nitrogenase activity by reversible ADP-ribosylation of the Fe-protein was first discovered in *Rhodospirillum rubrum* [70] and *Azospirillum lipoferum* [71] and shown to require a pair of genes, *draT* which encodes an ADP-ribosyltransferase and *draG* which encodes an ADP-ribosylglycohydrolase. A search of the genome of Ac-8003 for *dra*T and *dra*G orthologs revealed the presence of a pair of substantially overlapping potential *dra*G genes (Achr_23630, 23620) with identity to an ortholog in *P*. *aeruginosa* BWHPSA043. Neither gene is present in Av-DJ. Comparison against other DraG orthologs suggest that the apparent overlap is the result of a frameshift at approx bp 780 in Achr_23630 which may inactivate the protein. As we could find no ortholog of *dra*T in Ac-8003 this leaves the genetic basis mechanism of ADP-ribosylation in Ac-8003 unesolved.

### Immunity and resistance

*Ac*-8003 contains several sophisticated systems for resistance to viruses and mobile genetic elements including CRISPR arrays and associated *cas* genes, several restriction-modification systems (RMS) and capsular glycoconjugates.

#### CRISPR/CAS/CSE gene clusters

CRISPR sequences and their associated *cas*/*cse* genes are thought to provide acquired immunity to incoming nucleic acids and are present in about 40% of Eubacteria [72]. The chromosome of Ac-8003 encodes 2 CRISPR sequences. CRISPR 1 (at ca 1,636,245 to 1,639,045) comprises 41, 28 bp repeats separated by spacers of 32 to 34 bp. Spacers are thought to be derived from fragments of incoming viral or other mobile genetic elements. It is located 3’ to tandem *cas/cse* gene clusters both of which belong to sub-type I (E/ECOLI). The 15 *cas* /*cse* genes are arranged in tandem clusters (CAS1, Achr_14800 to 14860; CAS 2, Achr_14870 to 14930) 5’ to the CRISPR 1. CAS1 proteins show the highest degree of similarity (73 to 88%) to those encoded by genes from *Pseudomonas* species but lesser similarity (66 to 77%) to orthologs in Av-DJ. However, CAS2 proteins show the highest degree of similarity to the γ-proteobacterium Hdn1 and *Pseudomonas stutzeri* but not Avin-DJ. CAS2 is not followed by a CRISPR.

CRISPR 2 is located close to the origin of replication (at 95,915) and comprises 12, 28 bp repeats (different to those in CRISPR 1) separated by 32 bp spacers. The 8 *cas*/*cse* genes (Achr_750 to 820) associated with this cluster (CAS3) also belong to the sub-type I (E/ECOLI) family and encode proteins with the highest sequence identity to orthologs from *Acidovorax* NO-1 or *Halomonas* not Av-DJ. Surprisingly, the 28 bp repeat sequence is exactly conserved in the CRISPR in *Desulfatibaculum alkenivorans* AK-01 though the spacers show no similarities.

Av-DJ contains 2 *cas/cse* gene clusters and 3 CRISPRs which appear to be phylogenetically unrelated to those in Ac-8003. CRISPRs 1 and 2 lie 3’ to a type 1 E/ECOLI *cas/cse* gene cluster (Avin_17170 to17240) and comprise 2 repeat/spacers and 5 repeat/spacers respectively separated by two protein coding genes. CRISPR 2 and *cas/cse* genes (Avin_31630 to 31570) of the sub-type I-C/DVULG are followed by a CRISPR characterised by 20 repeat/spacer elements.

It appears that the *cas/cse*/CRISPR clusters of Ac-8003 and Av-DJ have been acquired independently through HGTs and have not been derived from an ancestral *Pseudomonas* lineage. It has been suggested that the number of repeat/spacer units in a CRISPR correlates with a history of the activity of that *cas*-CRISPR cluster and the continuing need to retain the spacer to protect against repeated viral or plasmid attack [73]. It is interesting to note the significantly higher number of repeat/spacer units in Ac-8003 than in Av-DJ, especially the unusually very high number in CRISPR 1, suggests that Ac-8003 may live in an environment which renders it more prone to attack from foreign nucleic acid.

#### Restriction and modification systems (RMS)

A striking feature of Ac-8003 is the complexity of its RMS. The genome potentially carries gene clusters for 1 Type III (*Ach*I) and 4 Type I (*Ach*II, AchIII, AchIV and *Ach*V) RMS. Genes for *Ach*I (Achr_290,320,330) and *Ach*II (Achr_26070,26100,26120, 26140,26150) are present on the chromosome and the genes for the remaining three Type I systems, *Ach*III (Achr_c290,300,320), *Ach*IV (Achr_c550 to 600) and *Ach*V (Achr_f1960 to 1990, 2030) are present on plasmids pAcX50c, pAcX50d and pAcX50f respectively. The multiplicity of RMS systems which appear to be present in the genome correlates with the complex DNA methylation pattern observed in Ac-8003. One motif involves an ^m4^C modification of an 8 bp sequence with 180° rotational symmetry typical of Type II RMS though not all potential motifs in the genome were found to be methylated. This may result from *Ach*I activity. The inner 6 bp are the recognition sequence for *Xho*I and protection of a subset of *Xho*I sites in genomic DNA has been observed experimentally (S. Theophilou; unpublished work). However, the restriction endonuclease and methylases encoded in this system show the highest identity to the *Pst*II Type III system.

Four distinct 12 bp Type 1 methylation ^m6^A type motifs were observed three of which show greater than 99.3% modification of the total motifs present in the genome. A fourth Type I motif is methylated in only 84.1% of occurrences in the genome. Amongst the Type I systems, the endonuclease subunit (R) and the methyltransferase (M) subunits encoded by the *Ach*II genes show greater than 80% identity to orthologs from *P*. *stutzeri* or *P*.*aeruginosa* whereas the specificity-determining subunits (S) show the greatest identity to orthologs in the cyanobacterium *Arthrospira platensis* C1 and the γ-Proteobacterium, *Xenorhabdus szentirmaii*. The R, M and S subunits of *Ach*III (on plasmid pAcX50d) all show a very high degree of identity (>87%) to orthologs from several *P*. *aeruginosa* strains. By contrast the R, M and S subunits of *Ach*IV show high degrees of identity to orthologs in *Burkholderiales* bacterium JOSHI_001. The *Ach*V gene cluster on pAcX50f closely resembles the well studied *Ecoprr*l Type 1C RMS especially given that a potential anticodon nuclease—encoding gene similar to the prr ribonuclease is located between the R and S genes. The prr nuclease forms a complex with the *Ecoprr*l RM system and is known to provide defence against phage T4 infection in *E*. *coli* [74]. The R, M and prr-like proteins show very high identity (~92%) to orthologs from *Burholderia cenocepacia* whilst the S subunit shows ~60% identity to a counterpart in a number of *Escherichia coli* strains. It is not possible to assign any of the Type I methylation motifs to any of the gene clusters in the genome. It would be necessary to clone and express each RMS cluster in a system where the methylation patterns could be identified unambiguously.

By comparison Av-DJ appears to have only a single Type III RMS in an operon which includes an adenine-specific methyltransferase (N4/N-6 family) (Avin_52230) and a restriction enzyme (Avin_52330) which is adjacent to a gene encoding a putative phage integrase (Avin_347467). The genome of Av-DJ contains potential genes for four other DNA methyltransferases two of which are predicted to be cytosine specific (Avin_36050, 51690) and two adenine-specific (Avin_24470, 52350). However, genes for other restriction enzymes were not found.

#### Cell Surface and excreted polymers

Surface and excreted polymers play a major role in immunity against bacteriophage attack and predation, resistance to environmental stress, communal behaviour, biofilm formation, surface colonization and infection. Azotobacters are capable of producing a variety of surface polymers including alginates, levans and cellulose.

Both A. chroococcum and A. vinelandii synthesize and export alginates which have been the subject of many studies. They are required for the formation of desiccation-resistant cysts [11,75] and proposed to play a role in protecting nitrogenase and potentially other O_2_-sensitive proteins from O_2_ damage [76]. Whereas Ac-8003 can produce copious amounts of gum, Av-DJ is a derivative of a non-gummy strain OP (also known as CA or UW), a variant of the original soil isolate (strain O).

The genetic basis for alginate biosynthesis is basically conserved in the two organisms. Both contain a large operon encoding 12 *alg* genes (*alg*D,8,44,K,E,G,X,L,I,F,A) (Achr_31180 to 31290: Avin_10970 to 10860) and *alg*C and *alg*F (Achr_38610, 29020; Avin_02910,10870). Genes shown to be involved in alginate regulation include *alg*R (Achr_36860; Avin_47610) and the *algU*,*muc*ABCD operon (Achr_29020 to 28980). However, in Av-DJ (and its parent strain CA) only the *mucABCD* genes seem intact (Avin_13700 to 13730) whilst the *alg* *U* gene contains a ~1 kb insertion mutation close to the N-terminal-coding sequence. This has been proposed to be the cause of the non-gummy phenotype in this lineage [41,77].

The structure of alginates is influenced by periplasmic (AlgG) and secreted bifunctional poly (β-D-mannuronate) C5-epimerases /alginate lyases (AlgEs) which convert β-D-mannuronate residues in the basic linear alginate polymer to α-L-guluronates. Av-DJ contains at least 7 *alg*E genes, 6 of which are tightly clustered (Avin_51170 to 51250). The multiplicity of the *alg*E genes has been suggested to be important for cellular differentiation [78]. Ac-8003 contains *alg*G and just 4 *alg*E genes 3 of which are contiguous but arranged in two operons. The products of two of these (Achr_39560, 39570) show the highest identity to AlgE7 in Av_DJ (Avin_51250) and the third (Achr_39550) to AlgE2 in Av-DJ (Avin_51180). It is unclear why Av-DJ contains so many AlgE genes but potentially they produce differences in alginate structure and composition in the two organisms. However, their relative lower occurrences in Ac-8003 neither prevents it from producing copious amounts of gum or affects its capacity for encystment.

#### Capsule lipopolysaccharides (LPS)

The presence and structure of LPS have been little studied in *Azotobacters*. Genes for the potential synthesis and export of LPS are located in several loci in the genomes of Ac-8003 and Av-DJ. LPS Locus 1 in Ac-8003 and Av-DJ is a highly conserved cluster of 18 genes extending over 30Kb (Achr_7070 to 6900: Avin_44680 to 44860) which appears to be involved in the synthesis of both the lipopolysaccharide inner and outer cores. LPS Locus 2 in Ac-8003 is a large cluster spanning 41.7 kb (Achr_17650 to 17960) which appears to be required for synthesis of an O-antigen. It encodes 32 genes arranged unidirectionally. This cluster bears a strong similarity to a cluster in Av-DJ (Avin_29890 to 30130) but includes a contiguous cluster of 7 additional genes not present in Av-DJ but which show the highest identity to orthologs from *Pseudomonas* sp. GM79. LPS Locus 3 in Ac-8003 comprises 13 genes arranged unidirectionally over 15.7 Kb (Achr_35630 to 35750). This also shows a high identity to a comparable cluster in Av-DJ (Avin_05410 to 05300) but includes a further 2 genes with highest identity orthologs from *Pseudomonas* species. Both organisms contain at least two further smaller gene clusters which appear to be involved in LPS synthesis or export. LPS locus 4 in Ac-8003 is conserved in both organisms and comprises 5 genes of which three (*lpt*CAD) encode the transport system for transferring the O-antigen-lipidA to the outer membrane (Achr_29830 to 29880; Avin_12800 to 12840). A fourth gene (*lpt*D) involved in transport of the O-antigen-lipidA to the outer membrane is located on LPS-Locus 5 which comprises a small operon of three genes which also contains the *sur*A gene for the efficient folding of extracytoplasmic proteins (Achr_4820 to 4850; Avin_46280–46800).

Ac-8003 has an additional large cluster of chromosomal genes potentially for LPS synthesis (LPS Locus 6). This entire region has all the properties of a gene island possibly for a more complex and alternative surface structure for Ac-8003 which appears to have been acquired through one or more HGTs. This region spans ~64 Kb and contains at least 38 genes (Achr_26520 to 26880). It lies immediately distal to an ortholog of *rfaH* (Achr_26880) which in Enterobacteria is known to encode a transcription antiterminator which exerts control of transcription of long operons encoding cell surface components e.g. LPS, exopolysaccharides, pili etc. Locus 6 and. Locus 6 includes; 13 type 1 or 2 glycosyl transferases, 5 methyltransferases, 6 genes for the synthesis of rhamnose including a contiguous cluster of *rfb*BDAC genes (Achr_26670 to 26700). It also includes a cluster of 6 genes for polysaccharide export including a Type 1 export system encoded by *eex*D,E1,E2,F (Achr_26850 to 26820) which have been proposed to be involved in alginate export in *A*. *vinelandii* [79]. Other genes include those potentially for the synthesis of GDP-mannuronate (Achr_26740) and a gene encoding undecaprenyl-phosphate galactosephosphotransferase (Achr_26520). The region has a %G+C content which is distinctly atypical for Azotobacters comprising a 23.4 Kb region (2,991,984 to 3,015,425) of 51.58% G+C and an adjacent region of 34.8 Kb (3,015,426 to 3,040,234) of 57.6% G+C. Within this region only a few predicted protein products show the highest degree of identity to orthologs in Av-DJ. These include undecaprenyl-phosphate galactosephosphotransferase (Avin_16230), the *eex*D,E,F gene cluster (Avin_16420 to 16460) and the transcriptional activator, *rfaH* (Achr_26880; Avin_15910). All the other genes show highest identity to genes from various Pseudomonads, other γ-proteobacteria, α-proteobacteria and even cyanobacteria.

A cluster of 27 genes potentially involved in the synthesis of cell surface components in Av-DJ is also located distal to the *rfa*H gene (Avin_15910). The *rfb*BDAC cluster for rhamnose synthesis (Avin_ 15920 to 15950) has a different evolutionary origin from that of Ac-8003 and lies distal to *rfaH* in the cluster which appears to terminate with 8 genes encoding a Type II transport secretion system (Avin_16070 to Avin_16170). Genes located between these clusters include several glycosyl transferases and a gene potentially encoding a 45.7 KDa paracrystalline S-layer protein (Avin_16040). It is possible that this is the S-layer protein forming a tetragonal surface array in *A*. *vinelandii*. The protein comprises tetramers of subunits of 60 KDa which are extracted from the cell in distilled water [80]. A gene encoding a S-layer protein of this kind does not appear to be present in Ac-8003.

#### Novel coiled-coil protein

Locus 6 in Ac-8003 contains a gene (Achr_26570) potentially encoding a large protein of 1217 aas with a long central coiled coil region of ~650 aas from residues ~ 250 to 900. Searches of the protein database show that the best matches (~35 to 40%) are the N and C terminal regions which show identity to the corresponding regions of a protein (PMI35_05964) from *Pseudomonas* Sp. GM78. The *Pseudomonas* protein also contains a central stretch of 120 aas which is predicted to form a coiled coil though it is significantly shorter than, and shows little overall sequence identity, to any part of the central region of the Achr_26570 protein.

These two proteins are possibly the first members to be identified of a new family of coiled coil proteins. Their structure comprises a relatively conserved N-terminal domain of 250 aas, a central region of variable length of between 120 and 650 aas which forms a coiled coil but is not highly conserved at the aa level, and a relatively conserved C terminal domain of ~330 aas. The Achr_26570 protein contains 4 long tandem repeats of 54 aas between residues 310 and 660. The nucleotide sequence for this region also shows striking multiple repeats which suggests that it has expanded by tandem sequence duplications. In broad structure, the Achr_26570 protein resembles SMC chromosomal segregation proteins [81] an ortholog of which is present in Ac-8003 (Achr_ 25310) but differs in two important respects. The N- and C-terminal domains appear to be unique and show no identity to SMC proteins [81] and the N-terminal domain also lacks the essential Walker ATPase domain. Also SMC proteins have a central region which is not a coiled coil and forms a hinge to allow the protein to fold into a Y shape. However, in both the Achr_26570 and PMI35_05964 proteins the coils appears to be continuous and therefore it potentially forms a dumbbell-shaped protein with a flexible “bar”.

#### Cellulose and Levan synthesis

The ability of Azotobacters to produce cellulose fibrils has been observed but little studied [82]. Accordingly, both Ac-8003 and Av-DJ chromosomes possess a conserved contiguous cluster of *yhj*Q, *bcs*A/B, *bcs*C, *bcs* D and *bcs*Z genes (Achr_35860 to 35900: Avin_05100 to 05060). These have been identified as necessary for cellulose synthesis in bacteria [83]. In well studied bacterial systems, cellulose formation is regulated by cyclic-di-guanosine monophosphate (c-di-GMP) which functions as a reversible activator of cellulose synthase. The level of c-di-GMP is modulated in response to environmental conditions by the opposing activities of diguanylate cyclase (DGC) and phosphodiesterase A [83, 84]. Both Ac-8003 and Av-DJ contain a number of genes which encode different forms of both enzymes. Various functions have been ascribed to cellulose production including cell-to-cell and cell-to-surface anchoring potentially leading to flocculation, and biofilm formation. It is possible that cellulose formation as with alginate production may play a role in the subtle calibration of responses to O_2_ required to enable N_2_-fixation in Azotobacters.

The ability of A. chroococcum including Ac-8003 to both produce and consume levans was discussed earlier (section on Polysaccharides).

Despite the multiplicity of potential barriers to phage infection seen in both Ac-8003 and Av-DJ, Duff and Wyss [39] showed that several strains of *A*.*vinelandii* and *A*. *chroococcum* including both of the now sequenced organisms were almost all susceptible to lysis by a number of different bacteriophage isolated from soil using *A*. *vinelandii* as an initial host.

#### Motility

Motility in bacteria can be achieved through a number of modes: flagella driven swimming, pilus-dependent twitching and gliding. Thompson and Skerman [7] showed that the possession of flagella was a common but not invariant characteristic of both *A*. *chroococcum* (16/19) and *A*. *vinelandii* (15/17). Ac-8003 was one of the few strains of *A*. *chroococcum* not possessing flagella but strains O and OP (the parents of Av-DJ) both possessed 1 or 2 flagella per cell. The genome of Ac-8003 contains only three chromosomally-borne contiguous genes (*flg*A,M,N: Achr_22620 to 22640) normally required for the synthesis of flagella. By contrast Av-DJ contains at least 42 genes for the synthesis of flagellae and the flagellum motor which are scattered in four loci. The largest cluster comprises 36 genes (Avin_23970 to 24330) not including the adjacent chemoreceptor encoding genes. This cluster starts with the *flg*A,M,N genes which show 68, 85, and 94% identities to the orthologs in Ac-8003. It appears likely that the rest of this large gene cluster has been deleted in Ac-8003. The other 9 genes in three loci in Av-DJ (Avin_27640, 27650; 27700; 27850 to 279200) are not present or have been deleted in Ac-8003. It is interesting to note that the Ac-8003 *flg*A,M,N genes are flanked by transposase genes which may have been involved in deletion events.

Whereas ancestors of Ac-8003 appear to have lost the capacity for flagella-driven swimming this strain does carry genes for twitching motility. This involves the extrusion and temporary anchoring of a specific pilus to an extracellular substratum, including fellow organisms and retraction of the pilus through a cytoplasmic membrane-located pilus retraction motor. This draws the bacterium closer to the anchor point, the anchor is released and the process is repeated [85]. The genetic components of this system in Ac-8003 occur in 6 loci, 5 of which are chromosomal. The major cluster of pilus genes *pil*GHIJK (Achr_38330 to 38920) is contiguous with *che*B and 2 *che*A orthologs (Achr_3280 to 3860) potentially encoding a chemotactic response regulator, and signal transduction system. Other genes potentially involved in twitching motility are distributed around the genome and include chromosomally-borne *pil*U, T1 (Achr_38420, 38430) and at least two other *pil*T-like pilus retraction motor genes (Achr_26460, 30440) and a further ortholog (Achr_d230) on plasmid pACX50d. All the twitching motility genes show the highest degree of orthologs in several *Pseudomonas* species especially *P*. *alcaligenes*. Whether these genes are sufficient to provide twitching motility for Ac-8003 is unclear but the possibility of twitching motility needs to be investigated physiologically. Av-DJ carries only *pil*G (Avin_03180) and a single *pil*T (Avin_03090) orthologs which suggests that ancestors of this organism may have possessed this capacity which has been subsequently lost.

#### Tellurite Resistance

In a study of the resistance of Azotobacters to a range of antimicrobials Thompson and Skerman [7]observed that 9/18 *A*. *chroococcum* strains including Ac-8003 were resistant to at least 0.001% w/v (~40 μM) potassium tellurite whereas none of 17 *A*. *vinelandii* strains were. Some strains of *A*. *nigricans* and *A*. *beijerinckii* were also shown to have similar levels of resistance seen in Ac-8003. Oxyanions of tellurium are highly toxic potentially causing the intracellular generation of reactive oxygen species superoxide radical [86] and depletion of glutathione and other intracellular thiols [87].

Ac-8003 contains at least 9 chromosomally-located genes possibly involved in tellurite resistance. Four of these are highly conserved in *A*. *vinelandii* and encode two TerB orthologs (Achr_32300, 10890; Avin_0985039420) and potentially four TerC orthologs (Achr_30780, 31590, 2740, 2830: Avin_11570, 10570, 49600) the latter of which may encode integral membrane proteins potentially involved in tellurite efflux. These four genes probably do not explain the greater tellurite resistance in Ac-8003 but this organism has a contiguous cluster of 4 genes *ter*ZAED/F (Achr_21170 to 21200) which are absent from Av-DJ. These genes are orthologs of four genes present in the tellurite resistance (Te^R^) operon (*ter*ZABCDEF) required for tellurite resistance in a number of bacteria [88] though the mechanism of this resistance is not yet understood.

### Other distinguishing characteristics of Ac-8003 and Av-DJ

#### Siderophores

The competition for acquiring Fe from the environment is a critical factor for growth of microbes and they have evolved repertoires of mechanisms for doing so. Many involve the synthesis and excretion of, often high affinity, Fe-binding siderophores to capture Fe and cognate cell membrane receptors and transporters to bind the siderophore and translocate Fe into the cell.

An invariant characteristic of *A*. *vinelandii* strains is their ability to excrete under Fe-limitation a diffusible yellow-green fluorescent siderophore, azotobactin, belonging to the pyoverdine family [89]. *A*. *vinelandii* stains are also known to produce a variety of strain-dependent catechol siderophores [90, 91].

Azotobactin synthesis is encoded within a large cluster of 12 (“*pvd*”) genes spanning at least 60 Kb (Avin_ 25550 to 25660). The cluster includes genes for: 4 large non-ribosomal peptide synthetases; ornithine 6-monoxygenase; a thioesterase, diaminobutyrate 2-oxoglutarate transaminase; a TonB siderophore receptor; a TauD/TfdA family transcriptional regulator and an iron-starvation PvdS-like Sigma factor. Gene products for catechol siderophore(s) synthesis and efflux in Av-DJ are encoded within a cluster of 8 (“*ent*”) genes (Avin_21150 to 21230). Mutagenesis of the *pvd* and *ent* loci confirmed the roles of these two gene clusters in ATCC 12837 [92].

Whilst *A*. *chroococcum* strains do not produce a fluorescent siderophore there are numerous reports of siderophore production by this species (e.g. hydroxamates by strain B-8 [93] and 184 [94]). The genome of Ac-8003 contains several gene clusters potentially involved in siderophore production. The largest comprises 19 chromosomal genes spanning 41 Kb encoding proteins (Achr_38860 to 39010) likely to be involved in the formation of a pyoverdine-like siderophore. The gene cluster includes: 4 non-ribosomal peptide synthetases; a polyketide synthase; a thioesterase; two efflux transporters; L-ornithine-5-monoxygenase; two TonB-dependent ferric siderophore receptors and a Sigma 70 factor. Apart for a gene encoding an MbtH-like protein (Achr_39010) none of the proteins encoded in this cluster show strong identity to any gene in Av-DJ. Instead, they show at least 60% identity to orthologs from a variety of β-proteobacterial species including *Janthinobacteria*, *Thauera* and *Variovorax*.

Ac-8003 contains two other loci which may be involved in peptide siderophore synthesis. One cluster of 8 genes (Achr_f2130 to 2200) is located on plasmid pAcX50f and will be discussed under that heading. A second cluster of 10 chromosomal genes (Achr_23750 to 23660) encodes a relatively small non-ribosomal peptide synthetase and several multidrug-resistance efflux pumps all of which show greatest identity to orthologs from the β-proteobacterial species *Pseudogulbenkia*. Two of the genes, one of which encodes a TonB-like siderophore receptor show the highest identity to orthologs in Av-DJ (Achr_23740; Avin_22830). Av-DJ but not Ac-8003 also contains an operon (*fhu*CDB) encoding a putative ABC-type Fe(III) transporter (Avin_50330 to 50350).

There do not seem to be any genes in Ac-8003 showing identity to the *ent* gene cluster in Av-DJ. This suggests that Ac-8003 does not make a catechol-type siderophore. However, it is interesting to note that Ac-8003 and Av-DJ both contain a highly conserved cluster of 5 genes with identity and identical gene order to the *pvs*ABCDE operon for the synthesis of the polyhydroxcarboxylate siderophore vibrioferrin in *Vibrio parahaemolyticus* [95].

### Pigment formation

*A*. *chroococcum* strains characteristically produce non-diffusible grey/black/brown pigments [7]. We have observed that colonies of Ac-8003 develop a yellow/brown colour when grown on nutrient agar but not on simple defined Burk’s-type media. These pigmentation patterns could be due to at least two types of compounds; melanins and carotenes. There is genetic evidence that both may be produced by Ac-8003. These secondary metabolites are multifunctional and the suggestion has been made that melanin formation by *A*. *chroococcum* may have a role in minimising oxidative stress [96] and carotenes are generally recognised to be antioxidants.

#### Melanins

Melanin formation has been studied in several different *A*. *chroococcum* strains (e.g. [96, 97, 98]. Melanin formation appears to be stimulated by the presence of copper in the medium which has led to the suggestion that a copper-dependent enzyme possibly a polyphenol oxidase (or laccase) may be required. Shivraprasad & Page [96] were able to measure polyphenol oxidase activity in cell extracts of *A*. *chroococcum* 184. Herter *et al*. [97] have suggested that melanin formation is dependent on a membrane-bound polyphenol oxidase in strain SBUG 1484. Ac-8003 contains 4 chromosomal genes potentially encoding polyphenol/multi-copper oxidases. Two potential *cum*A genes (Achr_10550, 30620) have orthologs with high degrees of identity in Av-DJ (Avin_39690, 11950) and lack export signal sequences. The other two genes are arranged as adjacent monocistonic operons. They encode type 2 multicopper oxidases both of which are predicted to contain an N-terminal signal sequence likely to direct the proteins to at least the periplasmic space. The two proteins (Achr_2720, 2710) show 73% identity to each other and 50% identity to orthologs in the γ-proteobacteria *Marinobacterium rhizophilum* and *Gallaeciomonas xiamenensis* respectively. We suggest that these proteins may be involved in melanin formation in Ac-8003 and corresponding proteins may be present in strains SBUG 1484 and 184.

#### Carotene synthesis

The chromosome of Ac-8003 contains a cluster of 7 genes (crtZ, E, idi, CrtX,B,Y,I: Achr_15260 to 15310) spanning 6.7Kb arranged into at least 3 operons. These genes potentially encode proteins e.g. CrtE (geranylgeranyl pyrophosphate synthetase), CrtB (phytoene synthase), CrtY (lycopene cyclase) and CrtI (phytoene desaturase) forming a pathway for the synthesis of a carotene-like compound. All these genes are absent in Av-DJ but show over 60% identity to orthologs in *P*. *stutzeri* where the gene organization is similar though not entirely conserved [99].

### Plasmids

A striking feature of Ac-8003 is the complexity of its genome which contains 6 plasmids ranging from 10.5 to 311 Kb in size. This complexity may be a common feature of *A*. *chroococcum strains* as indicated by earlier studies [40]. Plasmids have also been reported in other strains of *A*. *chroococcum* some of which have been associated with dissimilatory activities and resistance [53,100, 101]. All six plasmids appear to be low copy number. By contrast, Av-DJ contains no plasmids though a survey for plasmids in *A*. *vinelandii* showed single plasmids ranging in size from 13 to 78 Kb in 6 of 32 strains [102].

#### pAcX50f

Megaplasmid pAcX50f is a circular molecule of 311,724 bp containing potentially 292 protein coding genes and a number of non-coding RNAs including an ALIL pseudoknot and 3 cobalamin riboswitches (discussed further below). The nucleotide sequence and protein-coding regions are characteristic of *Pseudomonas* species. A nucleotide BLAST analysis shows that pACX50f has no overall identity to any other plasmid or replicon in the database. The plasmid carries core genes for replication initiation and terminus site-binding proteins and plasmid partitioning proteins. These include ParA, ParW (Achr_f1020, f1010), and PTRC B,E,C, B2 (Achr_f2100 to f2070), and PRTRC D (Achr_f1700). The plasmid also carries at least 8 *tra* genes, five in an operon comprising 6 genes (*tra*LEKBV: Achr_f1570 to f1620, a further two (*tra*ID) in a four gene operon (Achr_f1490 to f1520, and a monocistronic operon containing *tra*N (Achr_f1320). Several of these genes show highest identity to orthologs in IncF conjugative plasmids. It is unclear whether these genes alone are sufficient to enable self transmission. The plasmid also carries an operon of 4 genes encoding a Type I restriction enzyme and methylase (*Ach*V) (discussed earlier) and an *umu*DC operon which potentially encodes an SOS-controlled error-prone translesion DNA polymerase V which could be important for repairing thymine dimers formed by UV-irradiation.

The majority of genes in pAcX50f show the highest degrees of identities to those in *Pseudomonas* and *Burholderia species*. However, 57 genes (17%) in this plasmid show high degrees of similarity to proteins encoded in the chromosome of Av-DJ. They include a three gene operon (*gad*H1,H2,H3: Achr_f940 to f920) potentially encoding a membrane-bound 2-gluconate dehydrogenase and a contiguous cluster of genes including *ton*B *exb*B, *exb*D (Achr_f320 to f340) for an outer membrane transporter. The scattered distribution of chromosomal Av-DJ-like genes throughout pAcX50f suggest multiple inter-replicon interchanges within ancestral strains and/or the plasmid may have been assembled through a long history of HGTs between Pseudomonads and Azotobacters.

#### Corrin ring and B12 synthesis and requirements

**Fig 3:**
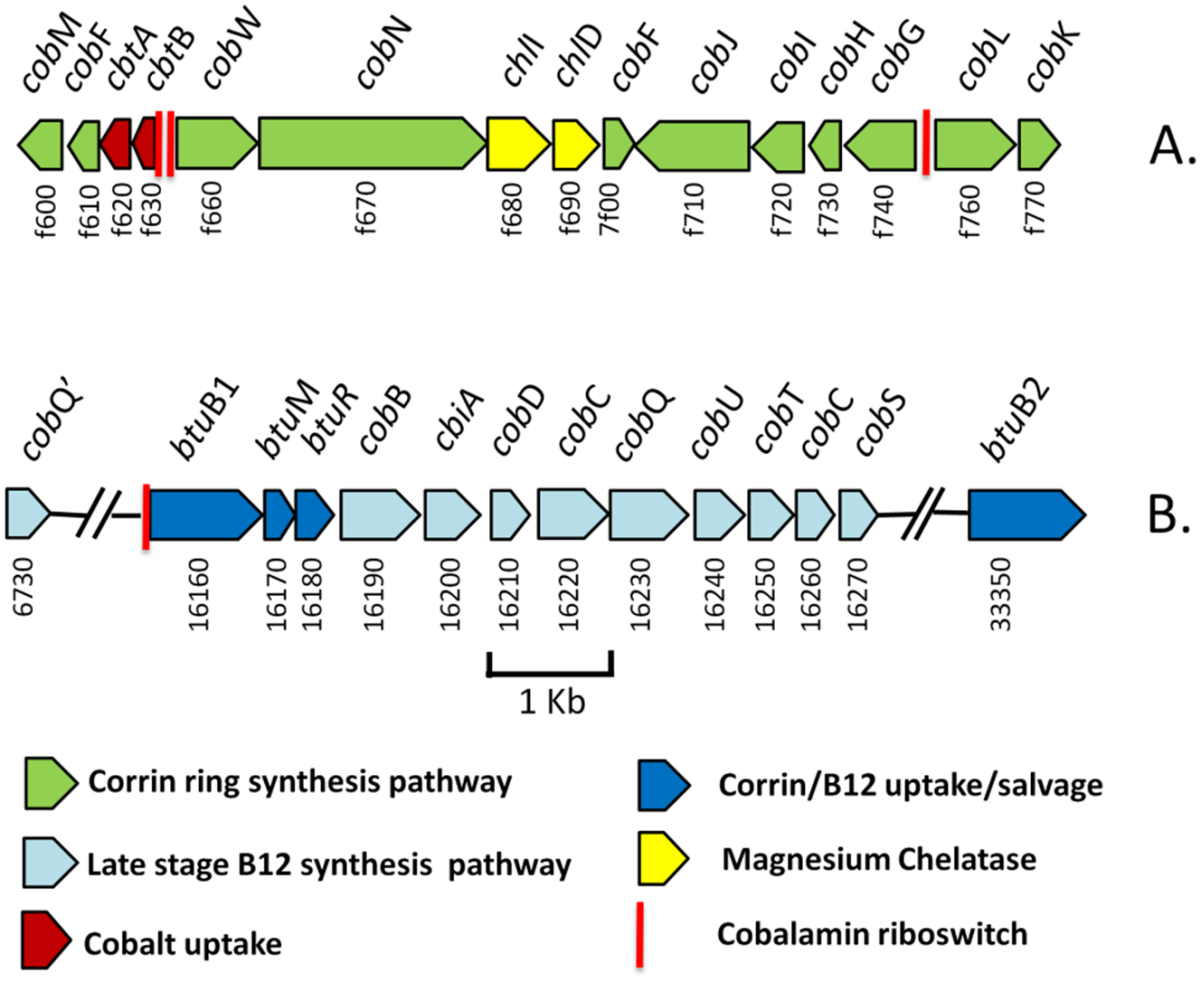
Plasmid- and chromosomal genes involved in Corrin and Adenosyl cobalaimin (B12) uptake, salvage and biosynthesis in *Azotobacter chroococcum* NCIMB 8003. A. The region of 16,967 bp from bp 64,013 to 80,225 in plasmid pAcX50f containing a contiguous cluster of genes for corrin synthesis. B. The regions of 12,882 from bp 1,777,467 to 1,789,349; 740,868 to 741,638 and 3,736,258 to 3,738,156 in the chromosome potentially containing genes for corrin and B12 uptake and salvage and the late steps in the synthesis of adenosyl cobalamin.

A notable feature of this plasmid is that it carries the genes for the entire 11 step aerobic corrin-ring forming limb of the Ado-CBL (B12) synthesis pathway extending from the synthesis of precorrin 2 to the late stage insertion of Co^2+^ into hydrogenobyrinic acid a,c-diamide [103]. The fifteen genes for this pathway (Achr_f600 to f770) are clustered in a region of 17 Kb and appear to be arranged into at least four operons separated by genes for three potential regulatory cobalamin riboswitches (Achr_f640, f650, 750) (Fig 3). These genes are absent from Av-DJ but show the highest degree of identity to orthologs from several *Pseudomonas* species though they are not all contiguously clustered in these organisms as in pAcX50f. The genes lie immediately adjacent to a gene potentially encoding a Tn or iTn which suggests that this gene cluster may be, or has been, a transposable element. The enzymes for the final steps in the formation of Ado-CBL and salvage of corrins and cobalamin from the environment are encoded by a chromosomally-borne twelve gene cluster in Ac-8003 (Achr_16160 to 16270). These are located immediately 3’ to a gene encoding a cobalamin riboswitch (Achr_16150). This gene cluster shows a high degree of similarity to the gene cluster in Av-DJ (Avin_33130 to 33030) but differs at the 5’ end by the absence in Av-DJ of *btu*M (Achr_16170). This is an optional component of a TonB-dependent vitamin B12 receptor encoded by the adjacent *btu*B gene (Achr_1616.) and the *cob*B gene for the salvage enzyme, cob(I)alamin adenosyl transferase. However, in Av-DJ BtuB is much larger than the Ac-8003 ortholog and appears to be a fusion protein in which the C-terminal 200 aas show 90% identity to CobB from Ac-8003. Both Ac-8003 and Av-DJ also carry a second chromosomal *btu*B gene encoding a potential TonB-dependent vitamin B12 receptor (Achr_33350; Avin_07860). These findings suggest that whilst Av-DJ and Ac-8003 are both able to salvage B12 and corrin precursors from the environment only Ac-8003 has the capacity to synthesize B12 *de novo* from tetrapyrrole precursors.

The potential value of the entire B12 pathway to Ac-8003 is presumably that it can always synthesise B-12 requiring enzymes when required. A comparative genomics study showed that Pseudomonads contain up to three B-12 enzymes [104]. The genomes of both Ac-8003 and Av-DJ carry chromosomal genes for all three. These are: *nrd*JaJb for the O_2_-independent ribonucleoside reductase (discussed earlier: Achr_570; Avin_00800); *met*H for the B12-dependent methionine synthase (Achr_18530; Avin_29240); and *eut*BC for ethanolamine ammonia lyase (Achr_32230, 32220; Avin_09920, 09930). However, neither obviously requires B12 for growth because they also carry genes for B12-independent counterparts/pathways: an O_2_-dependent Class 1 ribonucleoside reductase (*nrd*AB: Achr_15980,15970; Avin_33290,33300) and MetE for the B12–independent methioinine synthase (Achr_28820; Avin_13980). However, there appears to be no alternative pathway for the breakdown of ethanolamine. Thompson & Skerman [7] found that no Azotobacter tested could use ethanolamine as a sole C and energy source but it is possible that this compound could serve as a N source, the use of which we predict is likely to be B12–dependent in Av-DJ but not necessarily so in Ac-8003.

#### pAcX50e

Plasmid pAcX50e is a large circular molecule of 132,372 bp potentially encoding 111 proteins which shows no overall identity to any other plasmid or replicon in the database. The nucleotide sequence and protein-coding regions are characteristic of *Pseudomonas* species. The plasmid carries core genes for replication initiation and terminus site-binding proteins (Achr_e290, e280) and the partitioning proteins ParAB (Achr_e320, e330) and PRTRC BEC (Achr_e420 to e400). The plasmid carries a 6 gene operon encoding only three conjugation proteins TraI,D, ORF, ORF,ORF, TraL (Achr_e880 to e830). The plasmid “payload” potentially encodes a number of proteins nearly half (47%) of which show the highest degrees of similarity to orthologs from a wide range of *Pseudomonas* species. However, 31 gene products (21%) show high degrees of identity to proteins encoded in both the Ac-8003 and Av-DJ chromosomes. In particular, the 18Kb region of pAcX50e from 4,000 to 22,000 bp carries a cluster of genes which have duplicates scattered throughout the Ac-8003 chromosome. These include four pairs of genes for phosphate and especially polyphosphate metabolism: 2 exopolyphosphatases (Achr_e80, Achr_36790); 2 polyphosphate kinase-1s (PK-1s) (Achr_e90, Achr_36800); 2 polyphosphate kinase-2s (PK-2s) (Achr_e160; Achr_13560) and 2 Na^+^-dependent phosphate permeases (Achr_e170; Achr_13570). There are also orthologs of genes for fatty acid hydroxylases (Achr_e110 Achr_5020) and an alginate regulatory gene (AlgP: Achr_e100; Achr_140886). There are also orthologs of genes for mono- and disaccharide uptake/assimilation. These include a sucrose/maltose porin (*scr*Y-3: pAcX50e_940) of which there are two orthologs *scr*Y-1,2 in the chromosomes of Ac-8003 (Achr_40160, 39850) and Av-DJ (Avin_51790, 51510). The plasmid also encodes a glucokinase (Achr_e1000) which has two orthologs (*glk*1,2) in the chromosomes of Ac-8003 (Achr_27080, 37700) and Av-DJ (Avin_15690, 04130). pAcX50e also encodes a glucose/galactose transporter (*glu*P: Achr_e1010) whcih has two orthologs in the chromosome of Ac-8003 (Achr_37690, 39580) but one in Av-DJ (04150). Also present in this plasmkid is a sucrose isomerase gene (Achr_e950) with single orthologs in the chromosomes of Ac-8003 (Achr_33080) and Av-DJ (Avin_08330). A similar situation involves an operon encoding sulfoxide reductase (*yed*YZ) in the plasmid (Achr_e240, e250) which has orthologs in the chromosomes of Ac-8003 (Achr_33880, 33890) and Av-DJ (Avin_07350, 07340). It is unclear whether all these orthologs have arisen through vertical or horizontal transmission. For example, the three pairs of the plasmid/chromosomes orthologs for PK-2s, phosphate permease and fatty acid hydroxylase each show 99% identity which could have arisen by recent duplication in the Ac-8003 lineage or have been acquired via HGT from a closely related strain.

#### pAcX50d

Plasmid pAcX50d is a circular molecule of69,317 bp and encodes 55 potential proteins. The nucleotide sequence and protein-coding regions are characteristic of *Pseudomonas* species. It carries genes involved in plasmid replication (*repB*: Achr_d470), stable inheritance (*kfrA*) (Achr_d450) and toxin/antitoxin plasmid maintenance modules of the HipA, XRE and RelB/DinJ (*rel*BE: Achr_d500,d510) families. The other functions apparently carried by this plasmid appear quite disparate and potentially include a patatin/phospholipase-like protein (Achr_d100), an alkaline phosphatase (pAcX50d), a type I RMS system (*Ach*IV: discussed above), a diguanylate cyclase (GGDEF-domain), a zinc-dependent oxidoreductase and a site-specific phage-type recombinase (Achr_d210) The most numerous genes are 12 Tns/iTns from 6 families: IS4, Tn3, ISP1, IS630, ISXoo3, ISXo8. The plasmid contains a single conjugation gene (*tra*I: Achr_d460).

#### Plasmid pAcX50c

**Fig 4:**
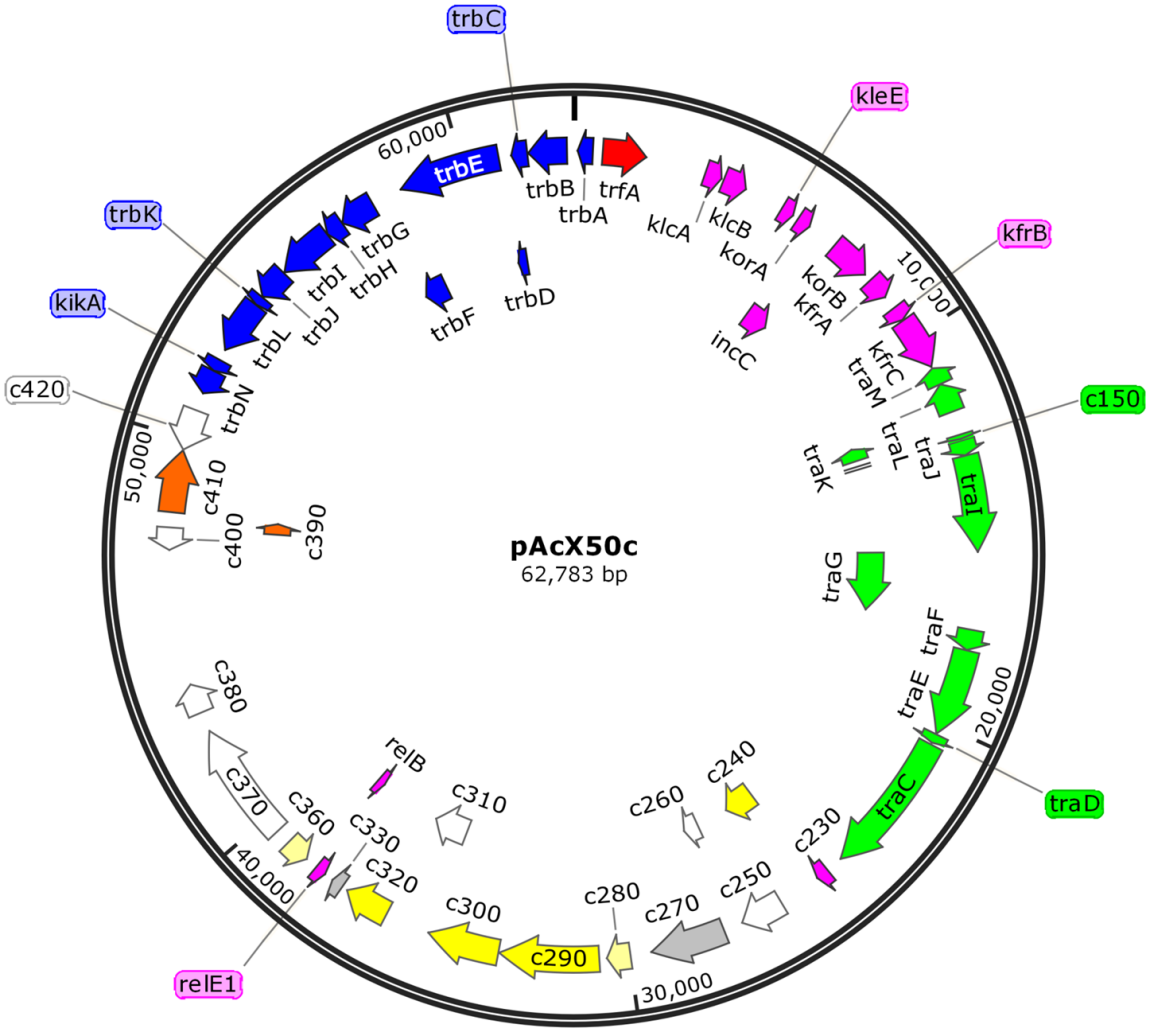
Physical and genetic map of plasmid pAcX50c from A. chroococcum NCIB 8003. The map shows the arrangement of deduced genes in the sequence of the potential retro-conjugative plasmid pACX50c. The two clusters of contiguous *tra* and *trb* conjugation genes are shown in green and dark blue respectively. Genes for other functions are indicated as follows: replication initiation (*trfA*: red); partitioning and maintenance (pink), Type I RMS system (*Ach*III) (yellow); transposases (brown); DNA binding and repair (grey); unidentified or conserved unidentified genes (white).

Plasmid pAcX50c is a circular molecule of 62,783 bp and carries 49 predicted protein-encoding genes. The nucleotide sequence and protein-coding regions are characteristic of *Burkholderia* species. It bears strong similarity to the IncP-1 group of broad host-range retrotransfer plasmids [105] which promote the conjugative transfer of genes from the donor to, and the capture of genes from, a recipient. Plasmid pAcX50c (see Fig 4) carries the core genes for replication *trf*A (Achr_c20) stable inheritance, partitioning proteins (*klc*ABC, *kor*A, *inc*C, *kor*B, *kfr*ABC: Achr_c30 to c110) and addiction (*rel*E1, *rel*B: pAcX50c340, c350) and almost all the core retrotransfer conjugation gene clusters typical of this plasmid group. These comprise the divergent *tra*JIGFEDC (Achr_c160 to c220) and *tra*KLM operons (Achr_c140-c120) separated by a *tra*J-II RNA gene (Achr_c150) and the *trb*BCDEFGHIJKL, *kik*A, *trb*N operon (Achr_c550 to c430, c10). The plasmid also contains the four gene operon for the Type I RMC (AchIII: discussed above). Studies of a large group of such plasmids which have been sequenced showed the presence of five distinct clades based on core gene sequence comparisons [106, 107]. It appears that pACX50c most closely matches plasmids isolated from *Pseudomonas* species amongst the γ-proteobacteria including pQKH54 [108]. In our earlier work pACX50c was apparently cured when the IncP-1 plasmid RP4 was introduced and stably maintained in the strain by selecting for the antibiotic resistance it encodes [40]. This would appear to provide further evidence that pACX50c belongs to the IncP-1 incompatibility group. These plasmids usually carry a “payload” of other genes often for antimicrobial resistance, catabolic functions or biocide degradation often flanked by transposases and which may in themselves constitute mobile genetic elements (MGEs). However, pAcX50c does not appear to carry any obvious payload of these kinds though it does carry the gene cluster for the Type I RMS system (*Ach*III) described above and several genes of as yet unknown function.

#### Plasmid pACX50b

Plasmid pAcX50b is a circular molecule of 13,852 bp which potentially encodes 10 genes. It contains a *trfA*-like replication initiator protein (Achr_b90), a *rel*EB operon encoding addiction/antitoxin proteins operon (Achr_b10, b20), a micrococcal SNase-like nuclease (Achr_b80). It contains two members of the abundant IS4 Tn family (ISAzch1) described earlier (Achr_b40, b70) and the remaining 4 potential genes include two non-coding RNAs, PrrB/RsmZ and a traJ-II (Achr_b50, b100).

#### Plasmid pAcX50a

Plasmid pAcX50a is a small circular molecule of 10,435 bp which appears to encode 9 potential genes one of which is a *trfA*-like replication initiator gene (Achr_a50) which shows 49% identity to an ortholog in plasmid pVEISO1 of *Verminephrobacter eiseniae* EF01-2, a member of the β-proteobacteria in the family *Comamonodaceae* and a nephridial symbiont of the Earthworm *Eisenia foetida*. All the other genes encode hypothetical or conserved hypothetical proteins.

## Conclusions

The genome of Ac-8003 is only the second member of the genus *Azotobacter* to be sequenced, the other being that of Av-DJ [41] and, more recently, strain CA from which Av-DJ was derived and CA6, a tungsten-resistant mutant of CA [42]. Whilst the complexity of the genomes is markedly different with Ac-8003 containing 6 plasmids and Av-DJ none, their chromosomes show significant synteny at the DNA level despite that of Ac-8003 being 14.3% shorter. The 2-dimensional plot of the alignment gives an X-shaped plot characteristic of exchange of significant regions across, and especially close to the points of replication initiation and termination thus broadly conserving gene replication order. Comparable intra-specific DNA alignments of the chromosomes of *Pseudomonas* species e.g. *P*. *stutzeri*, *P*. *mendocina*, *P*. *aeruginosa* and *P*. *fulva* show much weaker synteny and more diffuse X-plots. This suggests a relatively more recent divergence of the two Azotobacter species as compared to the Pseudomonad species which corresponds to the findings of phylogenetic analyses [9,10].

The structure of bacterial genomes can be described in terms of “core” and “pan” genomes where the core genome represents the genetic essence of the species or genus whilst the pan genome is the total of all genes so far identified in the species or genus. Those genes which are present in a species/isolate which are not core but which contribute to the pan genome have been considered variously as “non-essential”, “accessory”, “niche specific”, or “life-style” genes [109].

With only two *Azotobacter* genomes available it is premature to draw many conclusions about the general structure of *Azotobacter* genomes. Özen & Ussery [10] found that the genome of Av-DJ fitted well into the *Pseudomonas* group and when included the overall group core genome was reduced to 1,475 gene families in a pan genome of 29,626 gene families. From the two *Azotobacter* sequences now available it appears that the core genome for this genus can be reduced to approx. 3000 genes (Fig 2) whilst the pan genome is ~ 6,700 genes. There appear to be ~2000 accessory genes in Av-DJ which are not present in Ac-8003 whilst the latter contains ~1700 genes not found in Av-DJ. Of the accessory genes in Ac-8003, 1100 are chromosomal and the remainder plasmid-borne and the presence of a multiplicity of plasmids may also be an interesting feature adding significant diversity to *A*. *chroococcum* strains. As more sequences of this genus are determined, the core genome is likely to contract whilst the pan genome is likely to expand as found in a number of species or genera especially those which exist in diverse environments [110, 111].

The core genome of these two Azotobacters comprises the essential macromolecular architectures and machinery, and essential metabolic, anabolic and biosynthetic pathways. It also includes the genes which can now be identified as being required for the various common phenotypic characteristics in Azotobacters as reported in detail by Thompson & Skerman [7] e.g. the typical range of carbon and energy sources used. They also include the very highly conserved and well studied *nif* and *vnf* gene clusters for the Mo- and V-containing heterometal nitrogenases but not the *anf*-encoded Fe-only nitrogenase system. Also conserved with only minor differences are the genes for a multiplicity of terminal oxidases thought to be important for respiratory protection of nitrogenase and the membrane-bound NiFe-hydrogenase. The genome of Av-DJ was found to contain genes for several enzyme systems not suspected in Azotobacters namely CO-dehydrogenase, formate dehydrogenase and a soluble NiFe hydrogenase. These genes are also conserved in Ac-8003 suggesting that they serve an important role in the survival of both organisms in their distinctive natural environments. Av-DJ unusually contains two complete ATP-synthase operons which might be thought to be required to boost ATP synthesis for the highly ATP-requiring process of N_2_-fixation; however Ac-8003 contains only a single operon.

The accessory genes appear to fall into two broad groups. By far the largest group are those for which there are no obvious functional analogs in the other organism. The second group includes clusters of genes which are functionally analogous but appear to have been acquired through HTG rather than vertical transmission. This group can be sub-divided into at least two sub-groups. The first sub-group appears to comprise genes for “protection and defence” e.g. against phage attack and invasion by conjugative plasmids and other mobile elements. These include genes for capsule synthesis, restriction-modification enzymes and the CAS/CSE/CRISPR systems. These could also include a number of pilus producing genes both for conjugation and motility which may present a very different surface “canvases”. A second sub-group includes genes involved in the acquisition and salvage of essential elements, substrates and cofactors e.g. the gene clusters for the synthesis, efflux and recovery of Fe (and other metals) from pyoverdine-like high affinity siderophores. Whereas the cluster in Av-DJ is responsible for producing the conspicuous yellow green fluoresecent siderophore, azotobactin, its component genes products do not show strong sequence identity to putative pyoverdine genes in Ac-8003. The Ac-8003 genes show the highest sequence identity to orthologs from β-proteobacteria.

The phenotypic differences between Ac-DJ and Ac-8003 reported by Thompson & Skerman [7] probably reflect niche-specific activities. These can be correlated almost perfectly with the presence or absence of specific genes in the two genomes. A striking example noted by these workers was the presence of flagella in Av-DJ and absence in Ac-8003. The genomes show that Av-DJ contains many genes for flagella synthesis whilst only the *flg*AMN operon is conserved in Ac-8003 and the remainder appear to have been lost during evolution. This suggests that flagellum-driven motility is not of competitive advantage to Ac-8003 though other strains of *A*. *chroococcum* possess flagella. Instead the genomic evidence suggests that Ac-8003 but not Av-DJ may be able to move by twitching motility. These differences may reflect somewhat different niches that these two organisms occupy. *A*. *chroococcum* is common in soils where the ability to move through and across solid surfaces may be more advantageous and may also reflect the possibility that this isolate may have existed in soils of low water content (reviewed in [7]).

The two organisms typify their species in terms of the carbon and energy sources they can use. It is notable that Av-DJ is known to use the polyols; inositol, erythritol and glycerol and also aromatic compounds such as phenol. The genome sequence suggests it may also be able to use xylitol, arabitol, ribitol and sorbitol. By contrast Ac-8003 was only shown to be able to use mannitol and in our hands even this requires a period of “adaptation”. This may be because the organism appears to lack the genes encoding the putative ABC-type mannitol transporter and a mannitol dehydrogenase present in Av-DJ. It possible that mannitol may be taken up by other less efficient transporters. The potential to use benzoate also seems to be cryptic in Ac-8003 which is unusual for members of this species and may represent a “waning” function. By contrast Ac-8003 is known to be able to use polyglucans such as starch, glycogen and levans as carbon and energy sources. These functions seem to have been acquired by HGT from ancestors of current *Cellvibrio* species. These findings tend to suggest that *A*. *vinelandii* is adapted to use a greater range of more reduced C-compounds.

A highly distinctive feature of Azotobacters is their ability to form desiccation-resistant cysts. Whilst much is known about the role of lipids and complex polysaccharides in this process, it is not known whether any osmotic control accompanies this differentiation. This work identifies key proteins potentially involved in osmotic and cell volume control common to both Azotobacters which may be of interest in understanding how desiccation-resistance is achieved. It is interesting to find that Ac-8003 contains a group of genes involved with K+ acquisition and efflux which are known to play an important role in osmotic and cell volume control and which suggest that Av-DJ and Ac-8003 existed in distinctly different ionic environments e.g. one which was low in K^+^ in the case of Ac-8003.

A notable feature of the Ac-8003 genome is the presence of six plasmids, an unusually high number of extrachromsomal replicons. This confirms the plasmid profile observed experimentally [40]. All *A*. *chroococcum* isolates examined in that study contained a multiplicity of plasmids of which two contained plasmids which contribute ~600 and ~700 Kb to their respective genomes. Surprisingly five of the plasmids in Ac-8003 show no overall identity to any other plasmid in the public databases and therefore may be members of a group of plasmids which populate the Azotobacteraceae and may even be characteristic of *A*. *chroococcum* strains. One of the plasmids in Ac-8003, pAcX50c, belongs to the IncP-1 incompatability group and the genomic analysis shows it contains *tra and trb* gene clusters found in the IncP1-γ family of plasmids from Pseudomonads. Therefore, pAcX50c is highly likely to have conjugative and retroconjugative properties though this has not been tested yet. The remaining five plasmids have chimeric compositions with genes showing the highest identities to orthologs from various γ- and β-proteobacteria especially Pseudomonads and Burkholderias respectively. However, all the plasmids also carry a proportion of genes with the closest orthologs being to genes found in Azotobacter. These may have been acquired by inter-replicon exchange within the host or by horizontal transfer from other Azotobacters. Potentially these plasmids, especially the smallest two, pAcX50a and pAcX50b, could be exploited to develop cloning and expression vectors for this genus.

A striking characteristic of the Ac-8003 genome is the high number and variety of ISs/Tns. In general, such elements have been considered parasitic or non-essential, or of selective value under specific conditions. In a study of IS elements in over 100 bacterial genomes, Touchon and Rocha [73] concluded that genome size and, to a much lesser extent, ecological associations were the major factors that positively correlated with IS abundance in bacteria. Also, they concluded that IS families do not seem to be generally clade-specific and that the phylogenies of the various elements in a particular species do not align with its established phylogenetic position. This leads to the suggestion that IS elements are continually being acquired through HGT and also lost at potentially high rates. However, in Ac-8003 and Av-DJ and several Pseudomonads several elements appear to be clade specific or at least subject to frequent horizontal transfer between different members of this genus. Whether the high numbers of ISs/Tns in the two Azotobacters sequenced so far is a general characteristic of this genus will only become clear when the genomes of more members are sequenced. However, they may play a role in genome plasticity and evolution by promoting rearrangements, deletions and duplications which could be especially significant to Azotobacters [112].

The existence of 6 plasmids in Ac-8003 together with the great variety of other mobile elements suggests that in the course of its evolutionary history Ac-8003 has been subject to many “close encounters of a microbial kind”. It is generally accepted that whilst plasmids increase the load on the organism they can carry genes of conditional benefit to the host. More recently it has been suggested that they may carry genes for “goods and services” that also benefit other members of the community. Pools of genes which furnish those mutually available goods and services which are environmentally significant can be exchanged between various members of the community via the various modes of HGT. It has also been suggested that the full range of genes encoding “social traits” ranging from altruism through mutual benefit to selfishness and spite, can be carried by mobile genetic elements (summarised in [113]). For example, genes which encode proteins or compounds excreted by the organism can have both beneficial and detrimental impacts on community members. In this context it is interesting to speculate that the possession of genes for the entire corrin ring synthesis pathway in pAcX50f may benefit not only Ac-8003 but also organisms which need to salvage corrin/B12 from their environments. It is interesting to note that a naturally occurring vitamin B12-overproducing strain of *A*. *chroococcum* has been described [114]. It is also interesting to note that the presence of a B-12-dependent ribonucleotide reductase in both Ac-8003 and Av-DJ but in only 3 out of 8 *Pseudomonas* species [104] could enable the synthesis of deoxyribonucleotides especially in microaerobic conditions [115] which further supports the idea that Azotobacters are specialised for a microaerobic life-style [41].

Such “social” activities are unlikely to be restricted to mobile genetic elements. Also, the chromosomally-encoded abilities of Ac-8003 to break-down amylase, glycogen, to produce cellulose and levans, to produce several siderophores, to fix N_2_ (in [116]), and to be a reservoir of a wide range of mobile genetic elements constitute goods and services potentially available to other organisms. The production of viscous substances can influence the extent to which such goods and services can be exploited and clonal cooperation exists [117]). The apparently different modes of motility possessed by Ac-8003 and Av-DJ probably reflect the potentially different lifestyles of these organisms where surface adherence and social mobility is of selective advantage to Ac-8003 where a more aqueous environment may favour swimming behaviour in Av-DJ. The capacity for twitching motility has not been investigated in Azotobacters but is worth further exploration.

Several characteristics of some *A*. *chroococcum* isolates were not seen in Ac-8003 despite a careful search of the genome. For example there have been reports which have a long history of these organisms producing growth promoters such as indolyl-3-acetic acid (IAA) [118] and cytokinins [119]. We were unable to detect any ORFs in Ac-8003 that potentially encode enzymes known to be involved in the production of IAA in a number of organisms or for the production of cytokinins such as zeatin. Sindhy *et al*. [120] reported the isolation of a number of *A*. *chroococcum* strains which show multiple antibiotic resistances some of which have high levels of resistance. We were unable to identify any known antibiotic- resistance genes in Ac-8003.

Comparison of the Ac-8003 and Av-DJ genome sequences raises a wide range of interesting questions about the fascinating Azotobacter family. Our understanding of the evolution and diversity of these organisms and their place in the environment would benefit greatly from the availability of more genome sequences of other strains of *A*. *chroococcum* and *A*. *vinelandii* and examples of all the other defined species especially the plant-associated species *Azorhizophilus paspali*, the salt tolerant *A*. *salinestris* and of a member of the genus *Azomonas e*.*g*. *A*.*macrocytogenes*.

## Methods

For this study, Ac-8003 was obtained afresh from the NCIMB. It produces copious amounts gum on the standard N-free Burks’ medium with sucrose as C- source which can interfere with the preparation of genomic DNA. This was suppressed by growth on RM with mannitol (0.2%w/v) as sole carbon source and DNA was prepared as previously described [40].

DNA sequencing and methylation analysis was performed using the Pacific Biosystems (PacBio) Single Molecule Real Time (SMRT) sequencing platform and technologies. For SMRT DNA sequencing, as-isolated genomic DNA was used in order to retain the native methylation characteristics. A 20 kb library was prepared as follows. 5 μg DNA was sheared using a Covaris g-TUBE centrifuged at 5000 rpm for 1 min and concentrated with AMPure PB magnetic beads. Blunt ends were repaired and ligated to adapters according to the PacBio instructions. 20 kb fragments were selected and purified using BluePippin according to the manufacturer’s instructions. The annealing of fragments with primer, binding with Polymerase Binding Kit 5 and sequencing was carried out according to PacBio protocols. Base methylation characteristics were detected during SMRT using the unique kinetics of base addition observed opposite different modified template bases [121].

The sequence was assembled using the HGAP SMRT Analysis Software v 2.2.0 with a genome coverage of 110X. This technology produced unambiguous outputs for all 7 replicons without need for the construction of any gene libraries or any other gap closure methodologies. Coding regions and other features of the genome were identified using the Archetype Genomic Discovery Suite (Synthetic Genomics Inc). The detailed annotation pipeline underlying the Archetype software will be published at a later time. In brief, ORFs are identified (>30 aas using the standard genetic code) followed by gene flagging using a combination of *ab initio* methods such as Glimmer, Metagene and Prodigal as well as homology based approaches such as the use of Hidden Markov models such as PFAM, TIGRfam and BLAST Searches using the non-redundant database at NCBI [122]. Further refinement of gene starts was carried using GenMarkS [123].

The sequences of the chromosome and 6 plasmids have been deposited with Genbank under the accession numbers as follows: chromosome, CP010415; pAcX50a, CP010416; pAcX50b, CP010417; pAcX50c, CP010418; pAcX50d, CP010419; pAcX50e, CP010420; pAcX50f, CP010421. Supplementary material can be found at http://azotobacter.craic.com.

No ethical approvals were required in the pursuance of this work.
